# Discovery of potential natural dihydroorotate dehydrogenase inhibitors and their synergism with brequinar via integrated molecular docking, dynamic simulations and in vitro approach

**DOI:** 10.1038/s41598-022-23006-1

**Published:** 2022-11-09

**Authors:** Asmaa Khairy, Hala M. Hammoda, Ismail Celik, Hala H. Zaatout, Reham S. Ibrahim

**Affiliations:** 1 Department of Pharmacognosy, Faculty of Pharmacy, Alexandria University, Alexandria, 21521 Egypt; 2 Department of Pharmaceutical Chemistry, Faculty of Pharmacy, Erciyes University, 38039 Kayseri, Turkey

**Keywords:** Drug discovery, Plant sciences

## Abstract

The critical function of dihydroorotate dehydrogenase (DHODH) in pyrimidine synthesis attracted a great interest throughout beyond decades. Inhibitors of human DHODH (hDHODH) have validated efficacy for remedy of many immunological diseases. Brequinar and leflunomide are examples of such compounds. However, most of such immunosuppressive medications suffer from a lot of side effects and accompanied by adverse metabolic disturbances and toxicities. So that, immunomodulation utilizing natural products received the attention of many researchers. In this study, computer-aided molecular docking, molecular dynamic (MD) simulations and biochemical testing were utilized to find new pharmacologically active chemical entities from natural sources to combat immunosuppressive diseases. More specifically, Glide docking was used for a structure-based virtual screening of in-house 3D database of compounds retrieved from some traditionally known immunomodulatory plants surveyed from literature. The top five scored plants were found to be *Zingiber officinale, Curcuma longa, Glycyrrhiza glabra, Allium sativum and Olea europaea*. In vitro hDHODH inhibitory assays illustrated the ability of *Allium sativum and* silymarin standard hits; specifically, silibinin, to significantly inhibit the hDHODH enzyme. Molecular docking and MD simulations revealed a strong binding of the discovered hits within the active site. Following that, the most promising hits were tested separately with brequinar in a fixed-ratio combination setting to assess their combined effects on hDHODH catalytic inhibition. The binary combination of silibinin and brequinar revealed that in this combination, brequinar could be utilized at a dose 9.33-fold less when compared to its single-use to produce 99% inhibition for hDHODH enzyme**.** These findings confirmed that this binary mixture is an excellent combination providing better therapeutic effects and lower side effects.

Immune system disorders are a noteworthy clinical issue since of their persistent nature, the related healthcare costs along with their predominance in youthful populations. Current treatments, like TNF*α* and cytokine antagonists, have appeared incredible guarantee in treating numerous of these diseases such as rheumatoid arthritis^1,2^. However, many of the modern therapeutic agents goal the terminal stage of inflammation and do not address the essential issues which might be accountable for the initiation and development of the autoimmune process. In most cases, this requires proceeded and in some cases life-long treatment, resulting in an expanded hazard of threatening and irresistible complications. Tackling those disorders at their source requires an expertise of the way the atypical immune reactions arise, how they are sustained, and the natural mechanisms utilized to suppress those responses in healthful individuals^3^.

Autoimmune diseases vary incredibly within the organs they influence and in their clinical signs, with a few being constrained to specific tissues and others being systemic or dispersed^4^. Since all biological processes are dependent on cellular metabolism, during autoimmune disorders, metabolic dysregulation plays a role in maintaining cell proliferation, migration, and differentiation. Metabolic enzymes are important targets for autoimmune medication development since they play such a crucial part in this process^5^. Pyrimidine nucleotides assume a huge part in cell proliferation since pyrimidines are needed for the biosynthesis of RNA, DNA, phospholipids, and glycoproteins, and are connected by phosphodiester extensions to purine nucleotides in double-stranded DNA, both in mitochondria and nucleus^6,7^. So that the utility of inhibitors of the de novo nucleotide biosynthetic pathways, important for the proliferation of residing entities, provides therapeutic possibilities for the remedy of autoimmune disorders, such as multiple sclerosis, rheumatoid arthritis and cancer^8,9^*.*

Dihydroorotate dehydrogenase (DHODH), situated in the mitochondrial inner membrane, is an iron containing flavin-subordinate enzyme and assumes an essential part in the de novo synthesis of pyrimidine^10^. In a redox reaction (Fig. 1), DHODH catalyzes the change of dihydroorotate (DHO) to orotate (ORO), which is a crucial step of enzymatic responses in the de novo synthesis pathway of pyrimidine^11^. The first portion of the reaction showing ORO oxidation by electrons transfer from DHO to the flavin mononucleotide moiety (FMN). In the second part of the reaction and after the separation of ORO from the enzyme, the resultant dihydroflavin mononucleotide (FMNH2) is regenerated by another cofactor, called coenzyme Q (ubiquinone).

**Figure 1:**
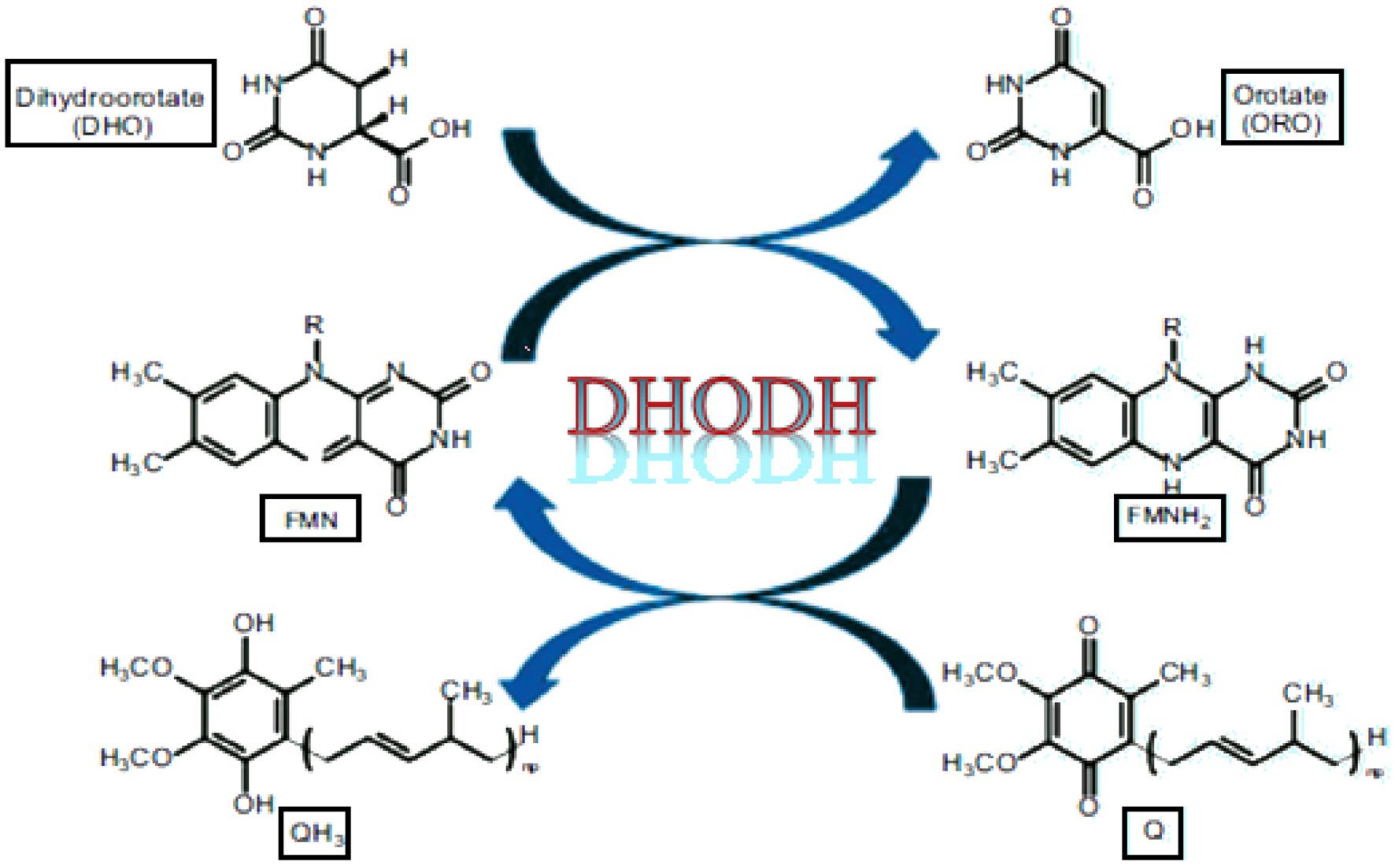
Schematic diagram illustrating reactions catalyzed by DHODH.

The critical function of DHODH in pyrimidine synthesis attracted a great interest throughout beyond decades^12^. Inhibition of DHODH results in decreased tiers of crucial pyrimidine nucleotides. Inhibitors of human DHODH (hDHODH) have validated efficacy for remedy of cancer^13,14^ and immunological diseases, including multiple sclerosis and rheumatoid arthritis^15–17^. DHODHs are appealing chemotherapeutic goals in diverse pathogens, including *Helicobacter pylori, Plasmodium falciparum, Enterococcus faecalis*^18–21^ and also as antifungal agents^22^. Human DHODH has some of residences that make it a specifically robust candidate as a brand-new drug target. Brequinar and leflunomide (Fig. 2) are examples of such compounds. Brequinar is an immunosuppressive and antitumor agent, whilst leflunomide indicates immunosuppressive activity^23–25^.

**Figure 2:**
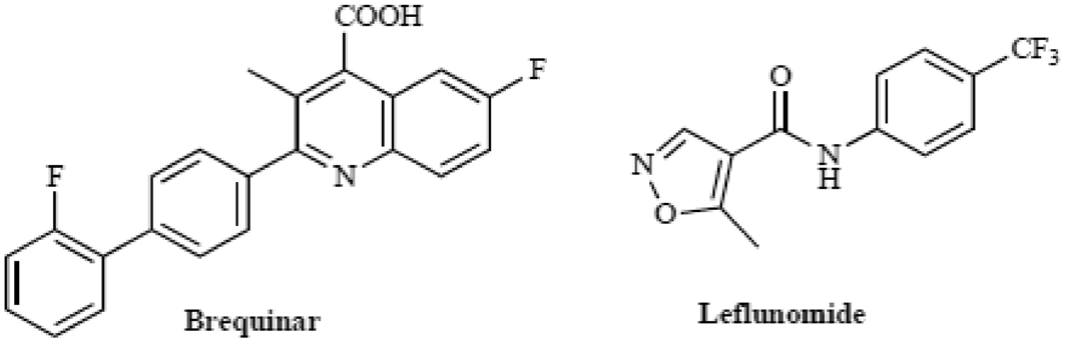
DHODH standard inhibitors.

However, most of such immunosuppressive medications have many disadvantages and suffer from a lot of side effects as it should be administrated for long term and accompanied by adverse metabolic disturbances and toxicities, in addition to the high risk of infection, cancer incidence and lack specificity^26–28^. For example, leflunomide is an intense immunosuppressant agent and has been used as an effective disease modifying anti-rheumatic drug (DMARD). However, it can cause serious haematologic, hepatic, dermatologic and respiratory adverse effects. There is a proof that leflunomide causes peripheral neuropathy, and introducing indications may mimic rheumatoid vasculitis^29^. Also, drugs in the brequinar class showed many side effects including leukocytopenia, thrombocytopenia and cellular depletion of bone marrow in addition to villous atrophy in jejunum^30^.

For the previously mentioned reasons, immunomodulation utilizing natural products received the attention of many researchers and can give an option in contrast to conventional chemotherapy. The capacity of some plants to hinder humoral and/or cellular insusceptible reactions can have helpful applications in some immune-mediated disorders^31^.

In this context, the objective of this study is to make a virtual screening of an in-house 3D database of compounds retrieved from some traditionally known immunomodulatory plants surveyed from literature. The top active hits based on in silico methods are to be further analyzed using in vitro assays for biological hDHODH inhibitory activity. Humam DHODH represents a novel target for screening natural phytoconstituents for alleviating autoimmune ailments. Finally, the study in hand deeply investigates, for the first time, the combined effect of top-scored immunosuppressive plant extracts and compounds resulted from the in vitro assays with brequinar on hDHODH enzyme. The results of this research will provide lead candidates from natural origin with potential safety and efficacy compared to the synthetic ones with lower production costs for managing autoimmune disorders.

## Results and discussion

### ADME and drug-likeness analysis

The ADMET analysis of an in-house database of 2154 compounds from 32 immunosuppressive plants (Table S2) was carried out using QikProp which anticipates some physiochemical characteristics such as the compounds solubility (QPlogS and CIQPlogS), molecular weights (mol_MW), number of hydrogen bonds accepted (accptHB) and donated (donorHB) to water molecules in medium. These four properties compose the Lipinski rule of 5 which determines the drug-likeness of the compounds where a compound that does not score more than 1 in Lipinski's rule would be considered active (Lipinski^32^). This rule provides information about the pharmacokinetics of the chemicals in living organisms. Results showed that 600 compounds of the database had an acceptable score according to Lipinski's rule (Table S2). In addition, the oral bioavailability of the gathered compounds was calculated. Only compounds of the database that obeyed the specified criteria were retained for further analysis.

### Virtual screening of phytoconstituents database and molecular docking analysis

A novel virtual screening strategy was reported in this research to develop new natural products that can inhibit the catalytic activity of hDHODH. The generated in-house database consisted of 2154 natural phytochemical components from 32 selected immunomodulatory plants (Table S1) together with the reference drug, brequinar. Since multiple crystal structures with high resolution for hDHODH, both with and without ligands, have been published, molecular docking has been considered as a promising strategy for virtual screening. The major target crystal structure chosen to imitate in vitro tests was that of hDHODH in complex with a leflunomide derivative inhibitor 4 (PDB ID: 3G0U) with the greatest resolution (2.0 Å).

Human DHODH tertiary structure is made up of two domains joined by an extended loop: a huge *C*-terminal domain which is composed of (MET78-ARG396) residues and a small *N*-terminal domain compiled of (MET30-LEU68) residues^33^. The *C*-terminal part is an assembly of an *α*-helices / *β*-strands with a central barrel of eight parallel *β*-strands encompassed by eight *α*-helices. The tiny *N*-terminal domain, utilized by the enzyme for its association with the inner mitochondrial membrane, comprises of two R helices and joined by a short loop.

From the tertiary topography of hDHODH, two sites were found to be accountable for its biochemical activity: (1) the redox site, composed of three antiparallel *β*-strands (C, D and E) at the head and two antiparallel *β*-strands (A and E) at the base, that acts as settling region for the dihydroorotate as substrate and flavin mononucleotide (FMN) restricting location as cofactor; (2) a tiny tunnel inside the *N-*terminus that allows the ubiquinone to quickly contact the FMN for completing the redox reaction. According to crystallography perspective, the narrow tunnel is the ultimate target for hindering the hDHODH action.

As a result, a better interpretation of the mode of putative hDHODH inhibitors activity requires more simplification of the *N*-terminus topography. The hydrophobic residues ALA55, GLN47, LEU42, LEU46, LEU58, LEU68, LEU359, MET43, PHE62 and PHE98 make up virtually entirely of the first tunnel's sub-site, which are engaged in membrane attachment as *α*_*1*_ and *α*_*2*_ helices pervious foundations. Two more sub-sites, made up of ARG136, GLN47, THR360 and TYR356, are located between the tunnel and the aforementioned redox site. The amino acids VAL143 and VAL134 form the last hydrophobic sub-site that caps the tunnel's narrow end^34^.

Docking analysis was carried out to investigate the best pose of chosen ligands with the objective protein to gain molecular perception into inhibitor’s mechanism of binding. Phytochemical constituents (2154) from 32 selected immunomodulatory plants along with one co-crystalized ligand, leflunomide and the reference drug, brequinar, were rated based on their extra precision docking scores.

As a result of the in silico screening experiments, this enormous database was filtered based on their total fitness scores. The plants with the largest number of active hits were chosen, and plant scores were calculated as illustrated in Eq. (1) in experimental section.

**Figure 3:**
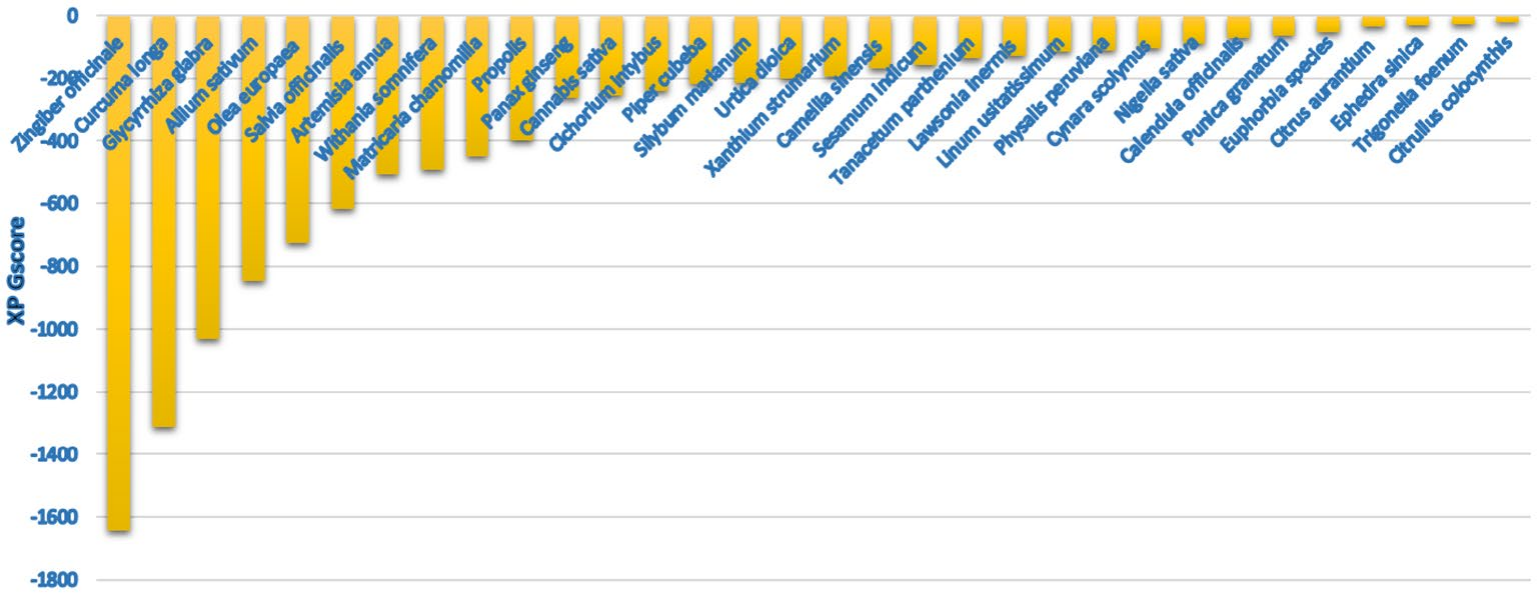
The overall in silico plants activity scores in descending order.

The screened plants were arranged based on their total docking scores (Fig. 3). The highest five active plants as shown by the virtual screening results were *Zingiber officinale, Curcuma longa, Glycyrrhiza glabra, Allium sativum and Olea europaea* with total XP Gscore scores of − 1643.133, − 1310.693, − 1032.134, − 848.969 and − 739.129, respectively. It is worthy to mention that; this is the first investigation of the direct inhibitory impacts of medicinal plants extracts on hDHODH activity.

Additionally, docking results revealed that silibinin (silybin) had the lowest binding energy (− 14.857 kcal mol^-1^) and the most favorable docking poses followed by silychristin B (− 14.642 kcal mol^-1^) and then 27- *O-β* -D-glucopyranosyl viscosalactone B (− 14.293 kcal mol^-1^) as shown in **(**Table 1). The free binding energies of the top 20 scoring hits and their plant source along with the co-crystalized ligand, leflunomide and the reference drug, brequinar, obtained through molecular docking with hDHODH crystal structure (3G0U), in addition to, types of binding interactions between ligands and critical amino acid residues in the hDHODH binding site are also depicted in (Table 1).

**Table 1:** Free binding energies of the top 20 scoring hits and their plant source along with the co-crystalized ligand, leflunomide and the reference drug, brequinar, obtained through molecular docking with hDHODH crystal structure (3G0U), in addition to, types of binding interactions between ligands and critical amino acid residues in the hDHODH binding site.

S. no	Name of top scored compounds (its plant source)	Binding free energy (Kcal mol^-1^)	Type of bindig interactions	Amino acid residues involved in protein ligand interaction
1		− 14.857	Hydrogen bonding (side chain)	TYR356, ARG136
			Polar interaction	GLN47, HIE56, THR360
			Hydrophobic interaction	LEU46, MET43, LEU42, PHE62, LEU58, PRO364, TYR38, LEU68, ALA59, LEU359, ALA55, PHE98, TYR356, TYR147, VAL143, VAL134, PRO52
			Pi-Pi stacking interaction	PHE62
			Glycine interaction	GLY363
			Charged (positive) ionic interaction	ARG136
2		− 14.642	Hydrogen bonding (backbone and side chain)	LEU359, TYR356, ARG136
			Charged (positive) ionic interaction	ARG136
			Pi-Pi stacking interaction	TYR38
			Glycine interaction	GLY363
			Hydrophobic interaction	LEU46, MET43, LEU359, PRO364, TYR38, LEU42, PRO69, LEU68, PHE62, LEU58, ALA59, ALA55, PHE98, TYR356, TYR147, VAL143, VAL134, PRO52
			Polar interaction	GLN47, HIE56, THR360
3		− 14.293	Hydrogen bonding (side chain)	ARG136
			Hydrophobic interaction	LEU46, MET43, ALA59, LEU58, LEU42, PHE62, TYR38, PRO364, LEU68, PHE98, LEU359, ALA55, PRO52, VAL143, VAL134, TYR356, TYR147
			Charged (negative) ionic interaction	GLU53
			Polar interaction	GLN47, HIE56, THR360
			Charged (positive) ionic interaction	ARG136
			Glycine interaction	GLY363
4		− 14.271	Hydrogen bonding (side chain)	ARG136
			Polar interaction	THR63, HIE56, THR360, GLN47
			Pi-Pi stacking interaction	PHE62
			Charged (positive) ionic interaction	ARG136
			Hydrophobic interaction	LEU359, PHE62, TYR38, LEU42, MET43, LEU46, ALA55, PRO52, VAL143, VAL134, TYR147, PHE98, TYR356, ALA58, LEU68, MET111, LEU58, PRO364, PRO69
			Charged (negative) ionic interaction	GLU53
			Glycine interaction	GLY363
5		− 14.267	Hydrogen bonding (side chain and backbone)	LEU42, THR360, ARG136, PRO52
			Polar interaction	THR45, THR360, GLN47, HIE56
			Charged (positive) ionic interaction	ARG136
			Hydrophobic interaction	PRO69, LEU68, TYR38, PHE62, LEU359, PRO361, ALA59, PHE98, ALA55, TYR356, VAL134, PRO52, LEU42, MET43, LEU46, LEU49,PHE37
			Glycine interaction	GLY363
6		− 13.951	Hydrogen bonding (side chain)	TYR356
			Polar interaction	THR63, HIE56, THR360, GLN47
			Charged (positive) ionic interaction	ARG136
			Hydrophobic interaction	TYR38, PHE62, MET111, PRO364, LEU68, LEU108, PHE98, LEU359, VAL134, TYR356, ALA55, PRO52, LEU46, MET43, ALA59, LEU58, LEU42
			Glycine interaction	GLY363
7		− 13.784	Hydrogen bonding (side chain and backbone)	TYR356, ARG136, THR360
			Polar interaction	HIE56, GLN47, THR360
			Pi-Pi stacking interaction	PHE62
			Charged (positive) ionic interaction	ARG136
			Hydrophobic interaction	LEU58, PRO364, MET111, LEU68, ALA59, LEU359, ALA55, PHE98, TYR356, VAL134, VAL143, PRO52, LEU46, MET43, PHE62, TYR38, LEU42
8		− 13.774	Hydrogen bonding (side chain and backbone)	TYR356, ARG136, ALA55
			Polar interaction	THR63, HIE56, THR360, GLN47
			Pi-Pi stacking interaction	PHE62
			Charged (positive) ionic interaction	ARG136
			Hydrophobic interaction	PHE62, LEU68, ALA59, MET111, LEU359, PRO364, PHE98, VAL134, TYR356, PRO52, LEU46, MET43, TYR38, LEU42, ALA55, LEU58
			Glycine interaction	GLY363
9		− 13.593	Hydrogen bonding (side chain and backbone)	ARG136, GLN47, PRO52
			Polar interaction	GLN47, HIE56, THR360, THR63
			Pi-Pi stacking interaction	PHE62
			Charged (positive) ionic interaction	ARG136
			Hydrophobic interaction	LEU46, LEU42, MET43, PRO52, VAL134, ALA55, TYR356, PHE98, LEU359, ALA59, MET111, PRO364, LEU68, TYR38, PHE62, LEU58
			Glycine interaction	GLY363
10		− 13.493	Hydrogen bonding (side chain and backbone)	GLN47, ARG136, PRO52, TYR356
			Polar interaction	GLN47, THR360, HIE56
			Charged (negative) ionic interaction	GLU53
			Charged (positive) ionic interaction	ARG136
			Hydrophobic interaction	LEU68, PHE62, LEU58, PRO364, ALA59, PHE98, ALA55, LEU359, PRO52, VAL134, VAL143, TYR356, LEU46, MET43, TYR38, LEU42
			Glycine interaction	GLY363
11		− 13.484	Hydrogen bonding (side chain and backbone)	TYR38, THR360, ARG136, PRO52
			Polar interaction	THR63, THR360, GLN47, HIE56
			Charged (positive) ionic interaction	ARG136
			Hydrophobic interaction	PRO364, TYR38, LEU46, PHE62, MET111, LEU68, LEU108, ALA59, LEU359, PHE361, MET43, PRO52, ALA55, PHE98, LEU42, TYR356, LEU58
			Glycine interaction	GLY363
12		− 13.376	Hydrogen bonding (side chain and backbone)	TYR356, PRO52, ARG136, THR360
			Polar interaction	THR63, HIE56, GLN47, THR360
			Pi-Pi stacking interaction	TYR38
			Charged (positive) ionic interaction	ARG136
			Hydrophobic interaction	PHE37, LEU58, MET111, LEU359, ALA59, PRO364, ALA55, PHE98, TYR356, PRO52, VAL134, LEU46, MET43, LEU67, PHE62, LEU42, LEU68, TYR38, PRO69
			Glycine interaction	GLY363
13		− 13.363	Hydrogen bonding (side chain)	ARG136
			Polar interaction	GLN47, THR360, HIE56, THR63
			Salt bridge interaction	ARG136
			Charged (positive) ionic interaction	ARG136
			Hydrophobic interaction	LEU42, PHE62, ALA59, LEU58, PRO52, ALA55, PHE98, TYR356, MET43, LEU359, MET111, PRO364, LEU108, LEU68, TYR38, LEU46, PHE37
			Glycine interaction	GLY363
14		− 13.052	Hydrogen bonding (side chain and backbone)	TYR356, ARG136, ALA55
			Polar interaction	GLN47, HIE56, THR360, THR63
			Charged (positive) ionic interaction	ARG136
			Hydrophobic interaction	ALA59, PHE62, LEU58, ALA55, PRO364, TYR38, LEU108, MET111, LEU68, LEU359, PHE98, TYR356, VAL134, PRO52, MET43, LEU42, LEU46
			Glycine interaction	GLY363
15		− 12.986	Hydrogen bonding (side chain and backbone)	TYR38, ARG136, ALA55, PRO52
			Polar interaction	HIE56, GLN47, THR360
			Charged (positive) ionic interaction	ARG136
			Hydrophobic interaction	PRO69, TYR38, LEU68, LEU46, PHE62, ALA59, LEU58, PHE98, ALA55, TYR356, PRO52, VAL134, MET43, LEU359, PRO364, LEU42,
			Glycine interaction	GLY363
			Charged (nagative) ionic interaction	GLU53
16		− 12.886	Hydrogen bonding (side chain)	TYR356, ARG136
			Polar interaction	THR360, HIE56, GLN47
			Charged (positive) ionic interaction	ARG136
			Hydrophobic interaction	PRO69, LEU68, PRO364, PHE62, TYR38, LEU359, ALA99, ALA55, PHE98, TYR356, TYR347, VAL143, VAL134, PRO52, LEU46, LEU42, MET43
			Glycine interaction	GLY363
			Pi-Pi stacking interaction	TYR38
17		− 12.822	Hydrogen bonding (side chain and backbone)	ARG136, THR360
			Polar interaction	HIE56, THR360, GLN47, THR63
			Charged (positive) ionic interaction	ARG136
			Hydrophobic interaction	LEU58, LEU46, MET43, ALA59, TYR356, ALA55, VAL134, PRO52, PHE361, LEU359, PHE98, PRO364, MET111, PHE62, LEU68, TYR38, LEU42
			Glycine interaction	GLY363
18		− 12.751	Hydrogen bonding (side chain and backbone)	ARG136, PRO52
			Polar interaction	THR63, HIE56, GLN47, THR360
			Charged (positive) ionic interaction	ARG136
			Charged (negative) ionic interaction	GLU53
			Hydrophobic interaction	LEU67, MET43, LEU46, ALA59, LEU359, MET111, PHE98, ALA55, VAL134, PRO52, TYR356, PHE62, TYR38, PRO69, PRO364, LEU68, LEU42, LEU58
			Glycine interaction	GLY363
			Pi-Pi stacking interaction	TYR38
19		− 12.724	Hydrogen bonding (side chain)	ARG136, TYR356
			Polar interaction	THR360, HIE56, GLN47
			Charged (positive) ionic interaction	ARG136
			Pi-Pi stacking interaction	PHE62
			Hydrophobic interaction	LEU58, TYR38, PRO364, LEU68, ALA59, LEU359, ALA55, PHE98, TYR356, VAL143, VAL134, PRO52, MET43, LEU46, PHE62, LEU42
			Glycine interaction	GLY363
20		− 12.688	Hydrogen bonding (side chain)	TYR38, ARG136
			Polar interaction	THR360, GLN47, HIE56, THR63
			Charged (positive) ionic interaction	ARG136
			Charged (negative) ionic interaction	GLU53
			Pi-Pi stacking interaction	PHE62
			Hydrophobic interaction	LEU42, TYR38, PHE62, MET43, TYR356, TYR147, VAL134, VAL143, PRO52, ALA55, PHE98, LEU359, MET111, LEU68, ALA59, PRO364, LEU58, LEU46
			Glycine interaction	Gly363
		− 10.449	Hydrogen bonding (side chain)	ARG136
			Polar interaction	GLN47, HIE56, THR360
			Pi-Pi stacking interaction	TYR38, TYR356
			Charged (positive) ionic interaction	ARG136
			Hydrophobic interaction	PRO69, PHE62, PRO364, LEU68, LEU46, LEU359, MET43, ALA55, PRO52, VAL134, VAL143, PHE98, TYR356, LEU58, ALA59, TYR38, LEU42
			Glycine interaction	GLY363
		− 7.649	Hydrogen bonding (side chain)	ARG136
			Polar interaction	GLN47, HIE56, THR360, THR63
			Charged (positive) ionic interaction	ARG136
			Pi-Pi stacking interaction	ARG136
			Glycine interaction	GLY363
			Hydrophobic interaction	ALA59, LEU359, VAL134, PRO52, TYR356, ALA55, PHE98, MET43, LEU46, PHE62, PRO364, TYR38, LEU68, MET111

**Figure 4:**
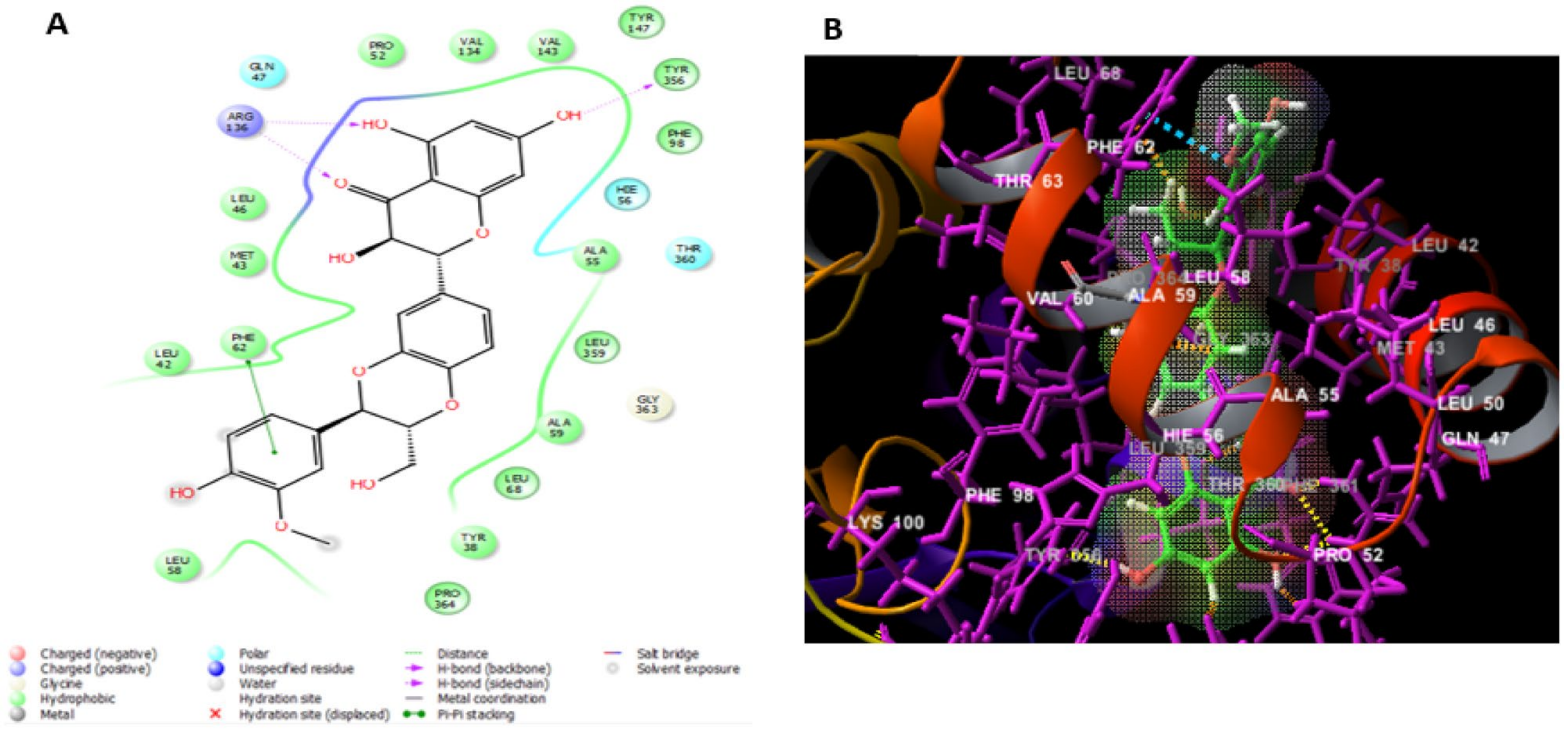
The docked pose of silibinin interacting with the amino acid residues of hDHODH active site in 2D (**A**) and 3D (**B**) sketches.

The top recognized molecule; silibinin interactions with the enzyme were illustrated in Fig. 4. Silibinin established two hydrogen bonds with TYR356, ARG136 residues, which are important for the enzyme tight binding. Additionally, Pi-Pi stacking interactions with PHE62 and numerous other hydrophobic interactions with LEU46, MET43, LEU42, PHE62, LEU58, PRO364, TYR38, LEU68, ALA59, LEU359, ALA55, PHE98, TYR356, TYR147, VAL143, VAL134 and PRO52 residues and also polar interactions with GLN47, HIE56, THR360 residues in a manner comparable to the binding modalities seen in the previously investigated enzyme substrates ensuring successful docking^34^.

**Figure 5:**
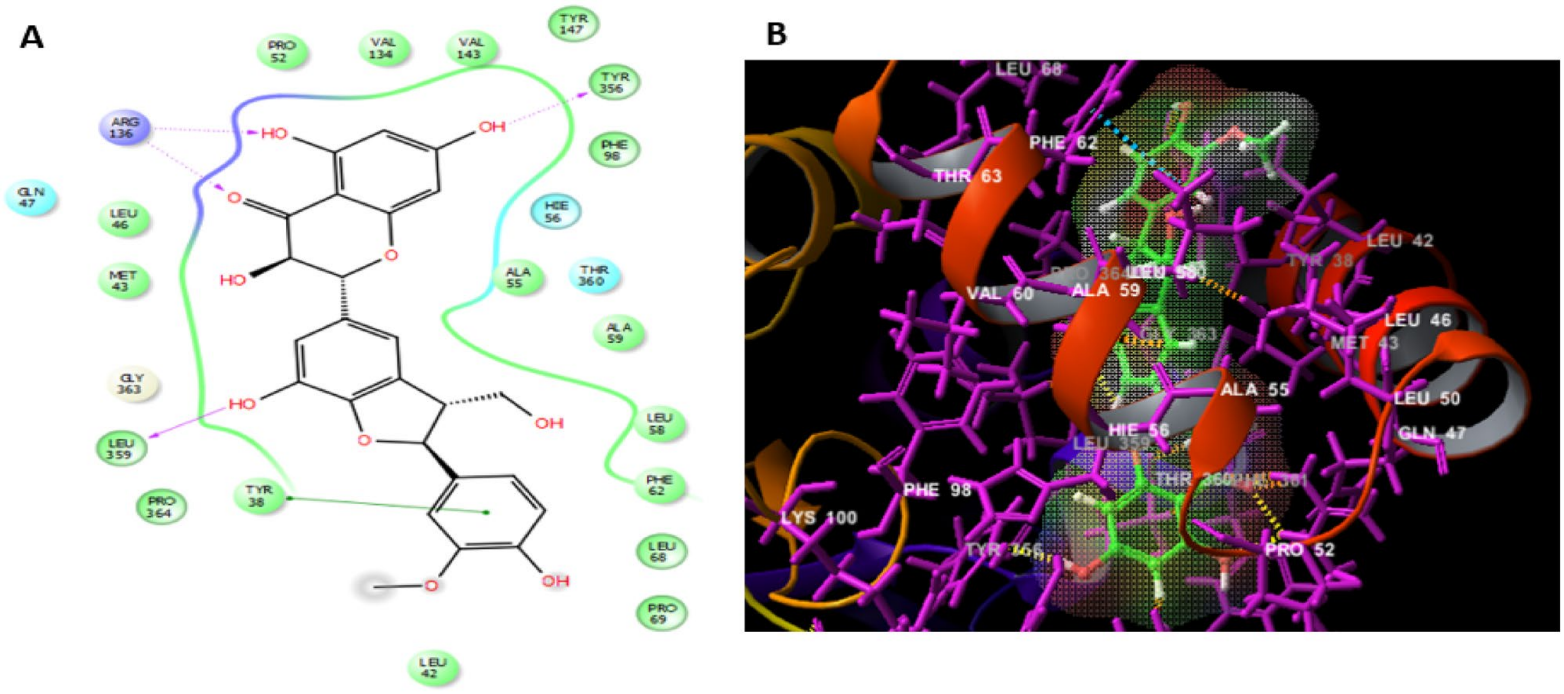
The docked pose of silychristin B interacting with the amino acid residues of hDHODH active site in 2D (**A**) and 3D (**B**) sketches.

Silychristin B is another natural flavonolignan constituent of silymarin, the standardized active extract of the fruit of *Silybum marianum*^35^. It is the second most abundant constituent in silymarin, after silibinin^36^ and was coming also in the second rank in our docking analysis after silibinin. Silychristin B formed the same interactions demonstrated in case of silibinin, except the π-π interaction with TYR38 residue instead of PHE62 demonstrated with silibinin, an additional hydrogen bond with LEU359 residue and one hydrophobic interaction with PRO69 residue as displayed in Fig. 5.

**Figure 6:**
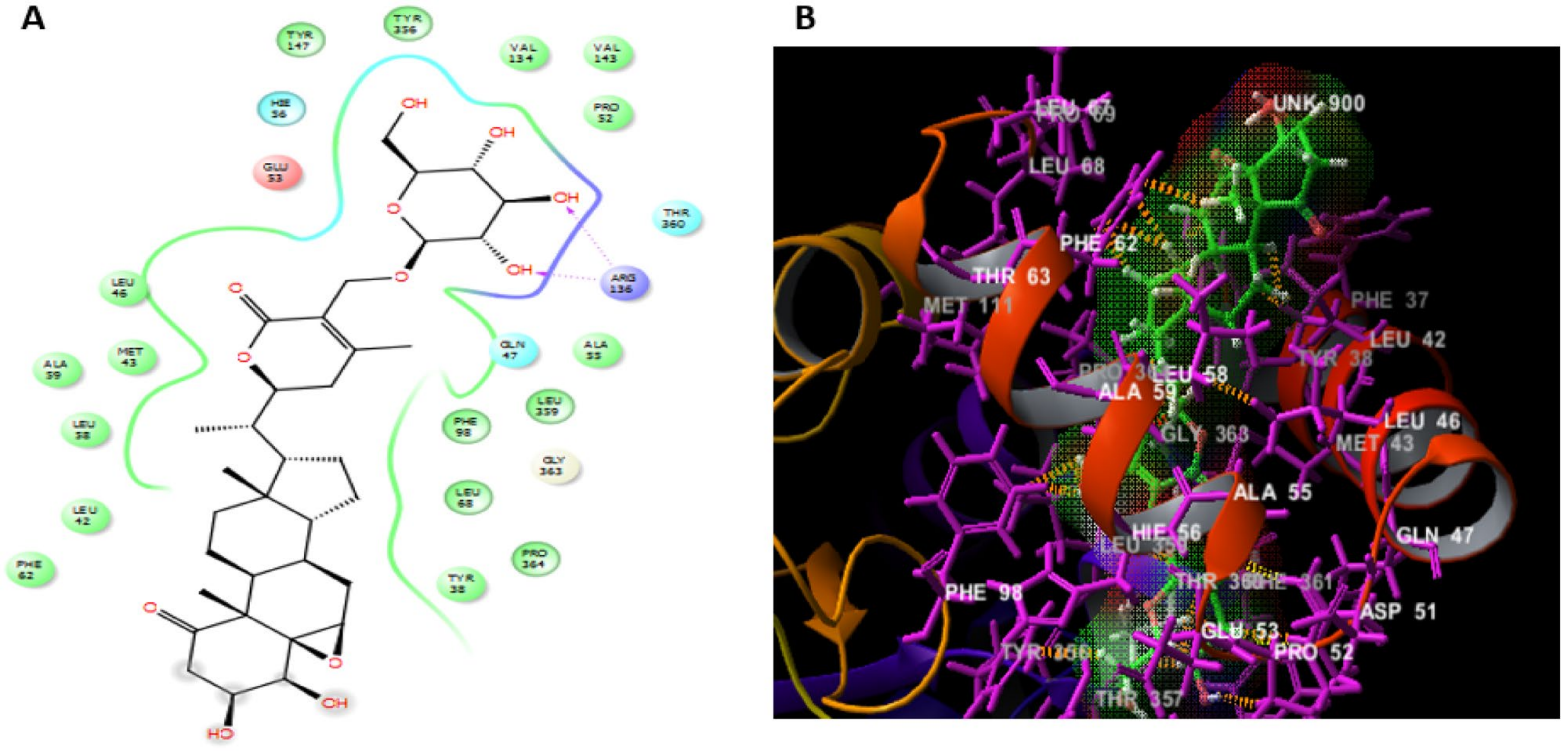
The docked pose of 27-*O-β-*D-glucopyranosyl viscosalactone B interacting with the amino acid residues of hDHODH active site in 2D (**A**) and 3D (**B**) sketches.

Moreover, 27-*O-β*-D-glucopyranosyl viscosalactone B which was ranked as the third compound of docking score, was found to be engaged with ARG136 residue with a hydrogen bond and GLN47, HIE56, THR360 amino acids residues by polar interactions. In addition to more hydrophobic interactions with LEU46, MET43, ALA59, LEU58, LEU42, PHE62, TYR38, PRO364, LEU68, PHE98, LEU359, ALA55, PRO52, VAL143, VAL134, TYR356 and TYR147 residues and negative ionic interaction with GLU53 residue as shown in Fig. 6.

The molecular docking analysis of two reference synthetic inhibitors of hDHODH, leflunomide and brequinar were also performed for comparison. These inhibitors are successfully positioned in the suggested ubiquinone binding site where polar and hydrophobic residues contribute to the binding. Brequinar exhibited binding energy − 10.449 kcal mol^-1^, whereas leflunomide revealed binding energy − 7.649 kcal mol^-1^ (Table 1). The inhibitors' carboxylic acid groups formed strong hydrogen bonding connections with ARG136 guanidinyl moiety and a polar interaction with GLN47 side chain as reported in previous literature^37^. These interactions were illustrated in Figs. S1 and S2 in supplementary materials. In comparison to the twenty listed naturally derived plant constituents, brequinar and leflunomide showed less interactions and lower binding free energy with the target protein.

### Molecular dynamics simulation

**Figure 7:**
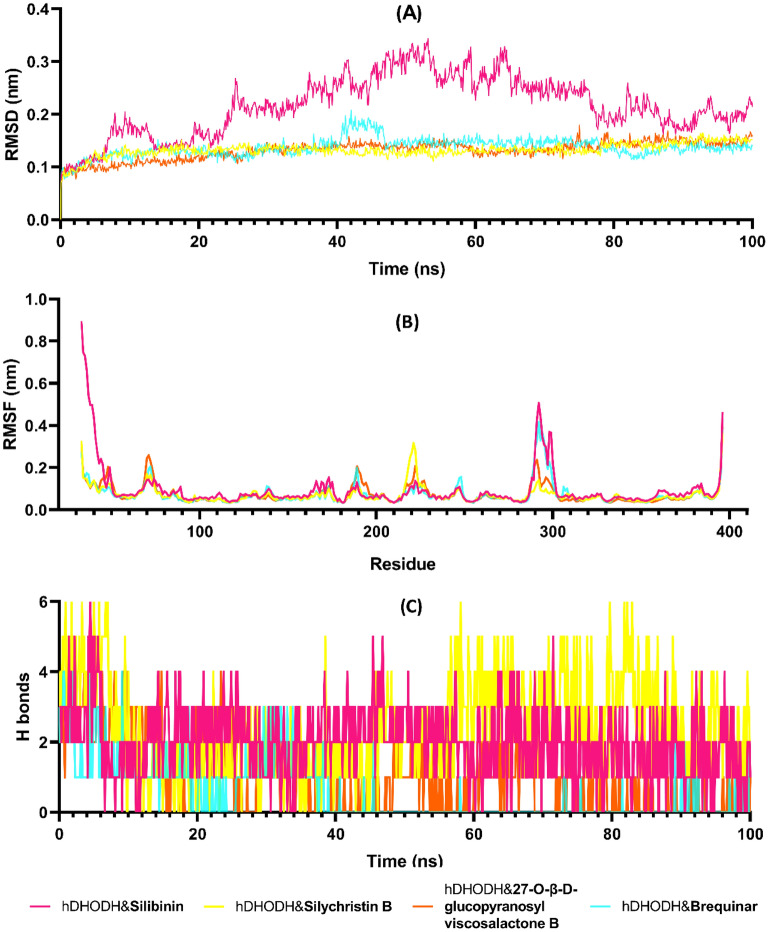
Molecular dynamics simulations trajectory analysis of hDHODH & silibinin, hDHODH & silychristin B, hDHODH & 27-O-*β*-D-glucopyranosyl viscosalactone B, and hDHODH & brequinar complexes for 100 ns. (**A**) The root mean square deviation (RMSD), (**B**) The root mean square fluctuation (RMSF) of protein–ligand complexes, and (**C**) Hydrogen bond number between protein and ligands during the molecular dynamics simulation.

Molecular dynamics (MD) simulations are used to predict and modeling how the interactions between macromolecules and small molecules occur in the physiological environment. MD simulation studies are used to investigate the stability and time-dependent variation of protein–ligand binding pose obtained from molecular docking studies. In this study, 100 ns duration and 1000 frames MD simulation of hDHODH & silibinin (electronic supplementary information video1), hDHODH & silychristin B (ESI video2), hDHODH & 27-*O-β*-D-glucopyranosyl viscosalactone B (ESI video 3) and hDHODH & brequinar (ESI video 4) protein–ligand complexes obtained from molecular docking studies were performed. Trajectory analyzes of RMSD, RMSF and H bond number changes were performed. RMSD is a parameter that provides information about the shifts in protein structure and stability. As shown in Fig. 7A, the hDHODH & brequinar, hDHODH & silychristin B and hDHODH & 27-*O-β*-D-glucopyranosyl viscosalactone B complexes were stable below 0.15 nm, while the hDHODH & silibinin complex was 0.3 nm up to 50 ns and then stabilized around 0.2 nm. RMSF analysis provides information on the flexibility and mobility of each residue during MD. As given in Fig. 7B, all but the hDHODH & silibinin complex fluctuated below 0.4 nm at the N- and C-terminals. A fluctuation of less than 0.1 nm was measured around the active site amino acids such as LEU42, GLN47, TYR356, and ARG136. Hydrogen bond interactions between protein and ligand are one of the most important interactions for strong bonding. In MD simulations, the H-bond remains stable over time and its number is maintained, which may imply a potent protein–ligand interaction. For this purpose, H bonds between hDHODH and compounds at 100 ns duration were analyzed. As given in Fig. 7C, at least one H bond formation is observed between the compounds and hDHODH. Silychristin B occasionally forms 6 H bonds, while silibinin often forms two to three H bonds.

**Figure 8:**
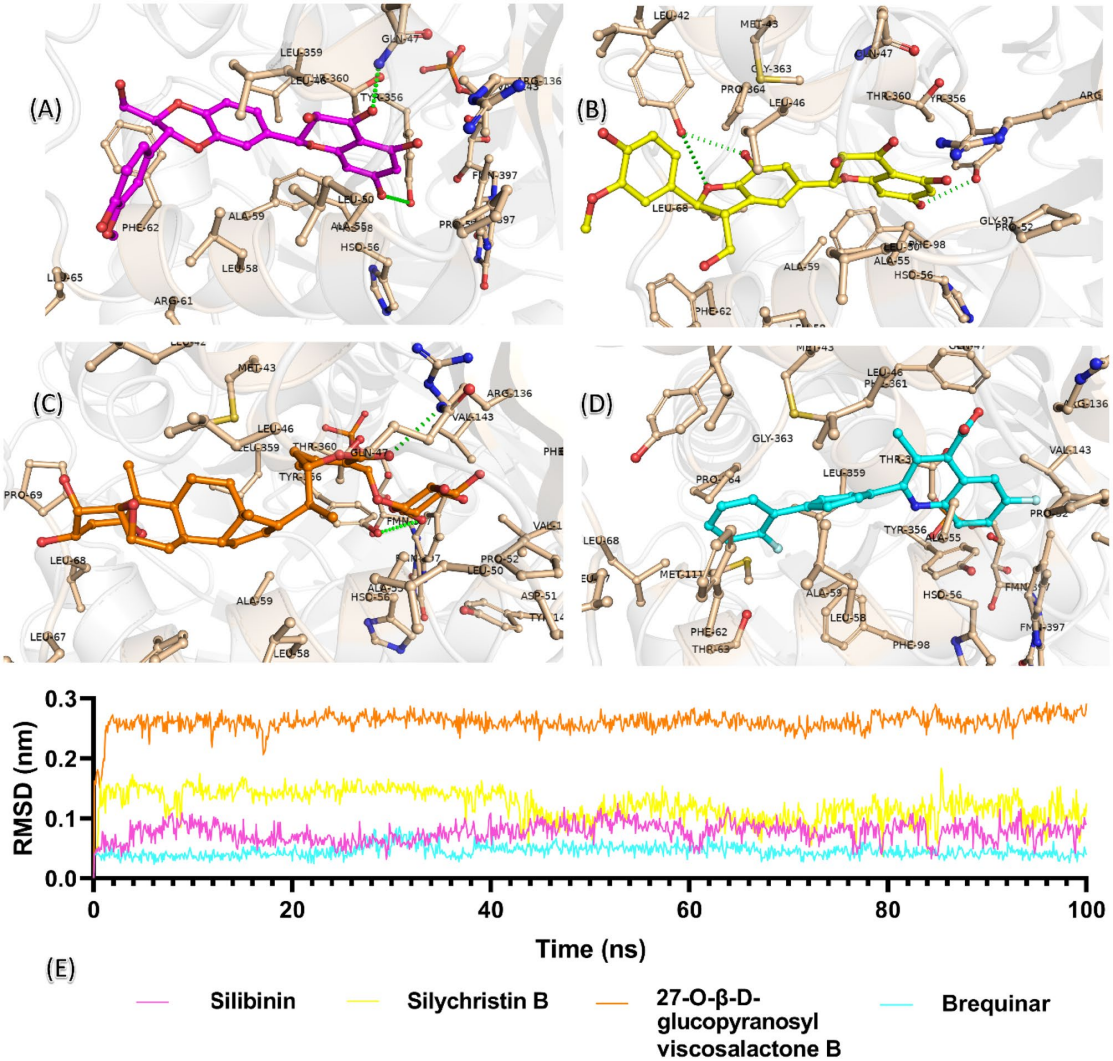
Protein–ligand interactions in molecular dynamics simulation of (**A**) hDHODH & silibinin, (**B**) hDHODH & silychristin B, (**C**) hDHODH & 27-*O-β*-D-glucopyranosyl viscosalactone B and (**D**) hDHODH & brequinar complexes at the end of 100 ns. (**E**) RMSD of compounds silibinin, silychristin B, 27-*O-β*-D-glucopyranosyl viscosalactone B and brequinar from the beginning to the end of 100 ns.

An analysis was performed to analyze the state of protein–ligand interactions over the 100 ns period. An animation video of the interactions of hDHODH & silibinin, hDHODH & silychristin B, hDHODH & 27-*O-β*-D-glucopyranosyl viscosalactone B, and hDHODH & brequinar protein–ligand complexes is given in the supplementary data. Accordingly, all four complexes remained stable at the active site. Also, protein–ligand interactions at the end of 100 ns were analyzed and the binding poses are given in Fig. 8. As given in Fig. 8A, silibinin has a 1.87 Å long H bond with GLN47 and formed hydrophobic interactions with cofactor FMN and active site residues LEU67, LEU55, ARG61, ALA59, LEU58, HIS56, ALA55, PHE98, TYR356, PRO52, LEU50, LEU46, ARG136, VAL143, and VAL134. The other compound, silychristin B, continued to interact with TYR356 in docking pose, and another H bond formation was observed with TYR38 (2.40 Å). As shown in Fig. 8B, it created hydrophobic interactions with PRO364, LEU42, MET43, LEU68, PRO69, THR360, GLN47, GLY97, PHE98, LEU50, LEU46, ARG136, ALA55, HIS56, LEU58, ALA59, and PHE62. Compound 27-*O-β*-D-glucopyranosyl viscosalactone B has a 2.09 Å long H bond with GLN47, FMN, PHE62, ALA59, LEU58, HIS56, PRO52, LEU50, LEU46, ARG136, VAL134, as given in Fig. 8C formed hydrophobic van der Waals interactions with MET43, LEU42, TYR356, THR360, LEU67, LEU68, PRO69. RMSD analyzes were performed from the trajectory to examine the motions of the compounds in the active region for 100 ns. As shown in Fig. 8D, brequinar formed van der Waals interactions with cofactor FMN, residues PRO364, GLY363, PHE98, MET111, THR360, LEU359, TYR356, VAL143, VAL134, ARG136, PRO52, ALA55, HIS56, LEU46, LEU58, ALA59, LEU42, MET43, TYR38, PHE62, THR63, LEU67, and LEU68. As described in Fig. 8E, the hDHODH active site in four compounds remained stable below 0.3 nm and their interactions continued. In particular, brequinar remained stable below 0.5 nm.

Another way to investigate protein–ligand interactions is to measure the binding-free energy MM -PBSA. In this study, MM-PBSA between ligand and protein was calculated from the average of the sum of van der Waals, electrostatic, polar solvation, and solvent accessible surface area (SASA) energies from 200 frames at 80 ns and 100 ns time intervals. As detailed in (Table 2), the average protein–ligand complexes of hDHODH & silibinin, hDHODH & silychristin B, hDHODH & 27-*O-β*-D-glucopyranosyl viscosalactone B and hDHODH & brequinar − 91.867 kJ/mol, − 99.817 kJ/mol, − 127.899 kJ/mol and − 115.470 kJ/mol MM-PBSA values were measured, respectively. According to the molecular dynamics analysis, all four compounds formed stable and potent interactions at the hDHODH active site.

**Table 2:** MM-PBSA binding free energies of hDHODH with compound silibinin, silychristin B, 27-*O-β*-D-glucopyranosyl viscosalactone B and brequinar between 80 and 100 ns.

Parameters Energy (kJ/mol)	Enzyme-ligand complexes			
	hDHODH Silibinin	hDHODH Silychristin B	hDHODH 27-*O-β*-D-glucopyranosyl viscosalactone B	hDHODH Brequinar
Van der Waals	− 198.722 ± 11.498	− 210.019 ± 17.584	− 246.627 ± 14.617	− 214.310 ± 11.383
Electrostatic	− 29.094 ± 10.789	− 46.316 ± 15.693	− 19.761 ± 8.491	− 21.325 ± 9.576
Polar solvation	158.823 ± 13.401	179.313 ± 17.302	167.251 ± 14.991	142.130 ± 17.439
SASA	− 22.875 ± 1.205	− 22.794 ± 1.221	− 28.762 ± 1.183	− 21.966 ± 0.791
Binding free	− 91.867 ± 12.201	− 99.817 ± 17.426	− 127.899 ± 16.599	− 115.470 ± 13.216

#### In vitro human DHODH inhibitory activity of top scored agents

Based on in silico screening results, additional laboratory-based in vitro screening of hDHODH inhibitory activity of the five-top scoring plants; *Zingiber officinale, Curcuma longa, Glycyrrhiza glabra, Allium sativum, Olea europaea* and *Salvia* officinalis in addition to the top scored compounds silibinin and silychristin B were performed utilizing spectrophotometric 2,6-dichlorophenolindophenol (DCIP) colorimetric test. 27-*O-β-*D-glucopyranosyl viscosalactone B couldn’t be tested due to its commercial unavailability. It was also found that silybin, silychristin B, neosilyhermin A, 2,3-dehydrosilybin A, isosilychristin, isosilybin and isosilandrin A which represent the components of silymarin possessed high rank in the virtual screening, hence standardized silymarin extract in vitro testing deemed crucial. The definitive goal of these in vitro investigations is to discover new agent approved as lead entity for the rational design of potential natural therapy with fewer or no side effects for the treatment of autoimmune disorders. Brequinar was employed as a positive control since it has been demonstrated to be a strong inhibitor of DHODH from bovine, rat, murine and human liver^38^. The utilized colorimetric in vitro method comprised dual examination in which oxidation of dihydroorotate and subsequent reduction of coenzyme Q6 are stoichiometrically equivalent to the reduction of DCIP which in turn is linked to drop in absorbance at 600 nm.

**Figure 9:**
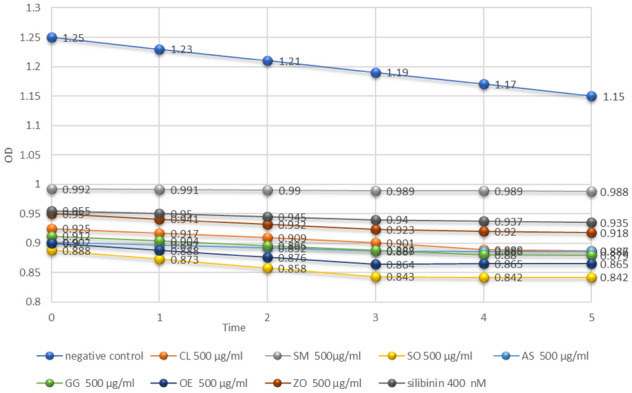
Time-absorption curve for *Curcuma longa* (CL) 500 ug/ml*,* silymarin standard (SM) 500 ug/ml*, Salvia officinalis* (SO) 500 ug/ml, *Allium sativum* (AS) 500 ug/ml*, Glycyrrhiza glabra* (GG) 500 ug/ml*, Olea europaea* (OE) 500 ug/ml*, Zingiber officinale* (ZO) 500 ug/ml and silibinin 400 nM.

**Figure 10:**
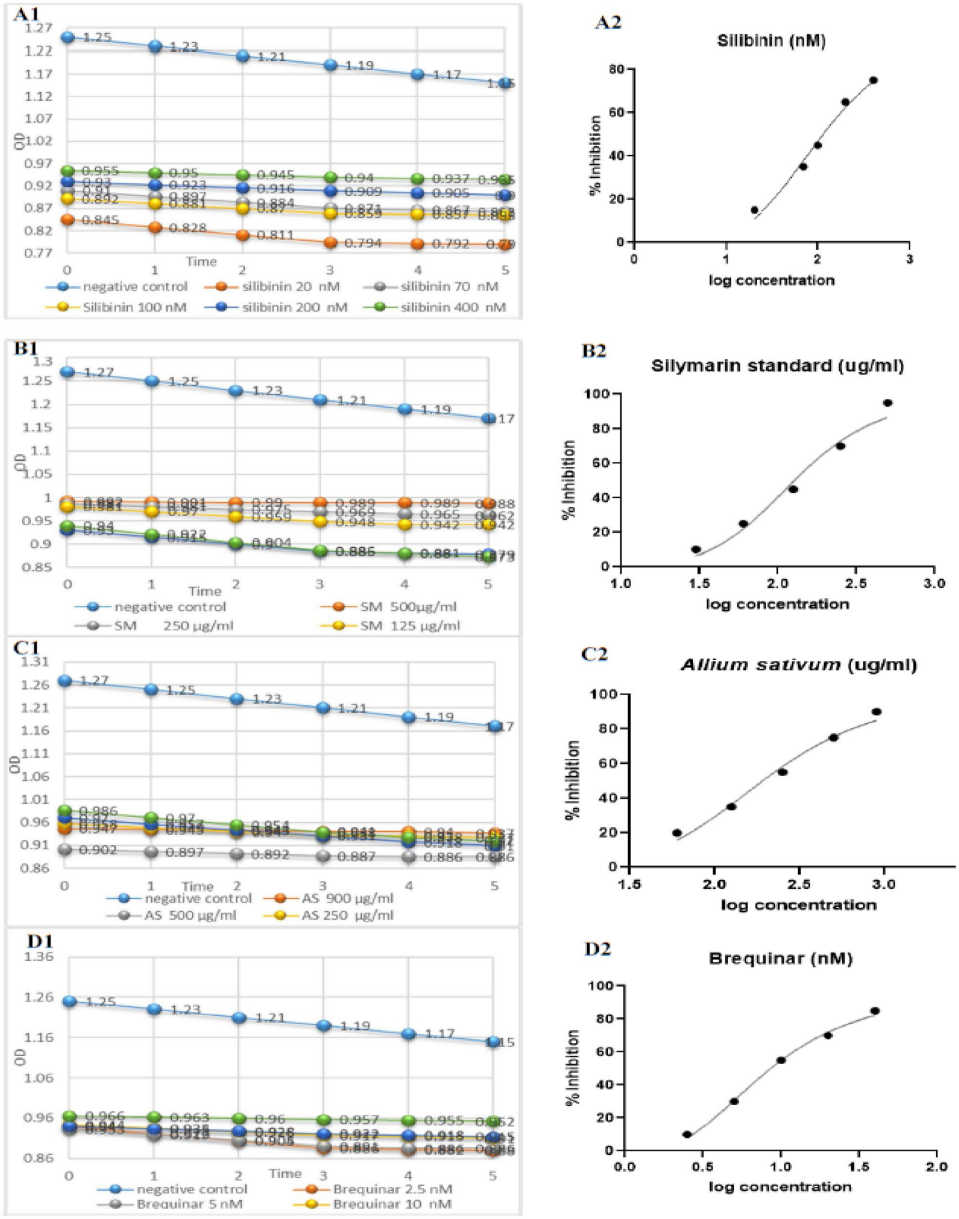
Time-absorption (to the left) and dose response (to the right) curves of silibinin (**A**), silymarin standard extract (**B)**, *Allium sativum* extract (**C**) and the reference drug; brequinar (**D**).

The hDHODH inhibitory activity for all tested agents was displayed as a decline in the optical density versus time as portrayed in Fig. 9. Their IC_50_ values together with brequinar were calculated by fitting the experimental data to a dose-response nonlinear regression curve utilizing Graphpad Prism (Fig. 10A–D**)**. Among the screened agents, silibinin (IC_50_ = 121.2 nM ± 0.014) was found to have the higher potency than silycristin B with IC_50_ value in the nanomolar range. The hDHODH inhibitory activity of other tested inhibitors was in the order of silymarin standard extract (IC_50_ = 133.2 ug/ml ± 0.031) followed by *Allium sativum* extract (IC_50_ = 201.5 ug/ml ± 0.044) (Table 3).

**Table 3:** In vitro hDHODH inhibitory activity of the tested agents.

The tested agent	IC_50_
*Allium sativum* extract	201.5 (ug/ml) ± 0.044
silymarin standard	133.2 (ug/ml) ± 0.031
Silibinin	121.2 (nM) ± 0.014
Silychristin B	166.1 (nM) ± 0.022
Brequinar	9.717 (nM) ± 0.063

*Data are demonstrated as mean of two tests ± SD.

#### The study of the synergistic inhibitory activity on hDHODH

On account of the high hDHODH inhibitory activity of *Allium sativum*, silymarin standard extract and silibinin as illustrated from their IC_50_ values, they were exposed to combination analysis with brequinar reference, the generally utilized immunosuppressive medication. The aim of these combinations is to take advantage of the opportunities for better therapeutic efficacy, diminished possible harmfulness of this synthetic drug, to postpone induction of drug resistance as well as looking for the best synergistic combination of brequinar with a natural immunosuppressive source. For this purpose, the combined inhibitory effect of *Allium sativum* extract, silymarin standard extract and silibinin with brequinar on hDHODH enzyme was determined.

#### The median-effect analysis approach for evaluating combined drugs interactions

A specific dose-effect curve depicting the accompanying parameters (Dm, m, and r) of each inhibitor singly and in binary combinations was produced according to Chou's hypothesis inferred from the median-effect principle^39^. Dm and m can be consequently calculated from the median–effect equation (Eq. 3 in experimental section) by utilizing compusyn program or indeed by employing a pocket calculator. Within the median-effect plot, Dm (practically equivalent to EC_50_) addressed the half maximum effective concentration needed to create 50% reduction in enzymatic activity. It could be estimated as the antilog of the x- intercept as represented in (Eq. 3.1) whereas m is the slope of the median-effect plot and r is the linear regression correlation coefficient.

As noticed in (Table 4), the Dm value for *Allium sativum* extract when combined with brequinar was less than the predicted additive impact of each individual drug, implying a modest degree of synergism at effect level 50%. In contrast, when silibinin and silymarin standard extract were coupled with brequinar, the Dm values were larger than the predicted additive impact of each individual drug, showing antagonism at the effect level 50%.

**Table 4:** Dose-effect curve parameters of *Allium sativum* extract, silymarin standard extract and silibinin individually and their binary combinations with brequinar.

Drug	Dose-effect curve parameters		
	Dm	m	r
*Allium sativum* extract	202.972 (ug/ml)	1.26701	0.99998
Silymarin standard extract	133.538 (ug/ml)	1.56510	1.00000
Silibinin	122.127 (nM)	1.00184	0.99999
Brequinar	9.76713 (nM)	1.33376	0.99999
*Allium sativum* extract + Brequinar	141.25 (ug/ml)	1.107	0.99889
Silymarin standard extract + Brequinar	135.453 (ug/ml)	1.027	0.99999
Silibinin + Brequinar	127.135 (nM)	2.2	0.98999

The boundaries Dm, m and r are the antilog of x-intercept, the slope and the linear correlation coefficient of the median-effect plot, respectively. CompuSyn was employed for automatic calculations.

Briefly, the Dm esteems for all individual entities along with their binary combinations were employed as a general reference point for forecasting antagonism or synergism at various effect levels using the CI (Eq. 3.1) and creating combination index (CI) plots^40^. In all cases, the (r-value) was more than 0.97, showing that the data followed the median-effect principle (Table 4).

### Analysis of Combination index (CI), Isobologram, and dose reduction index (DRI) for assessing drug-drug interactions

In expansion to pharmacological and molecular characteristics of the drugs, there are with no doubt other variables fundamentally affect the adequacy of medication combinations like drug concentrations, desired potency and drug ratios. Multiple strategies concerning the examination of combination effects including the analysis of the combination index, isobologram and dose reduction index (DRI) were used to demonstrate the extent and the kind of drug interactions with the goal of investigating the pharmacological relationships between *Allium sativum* extract, silymarin standard extract, and silibinin each with brequinar in order to develop effective drug combinations that could essentially progress quiet results in case of immunosuppressive diseases. It was noticed that these strategies provided complimentary data and yielded comparable conclusions.

### Analysis of *Allium sativum* extract and brequinar binary mixture

**Figure 11:**
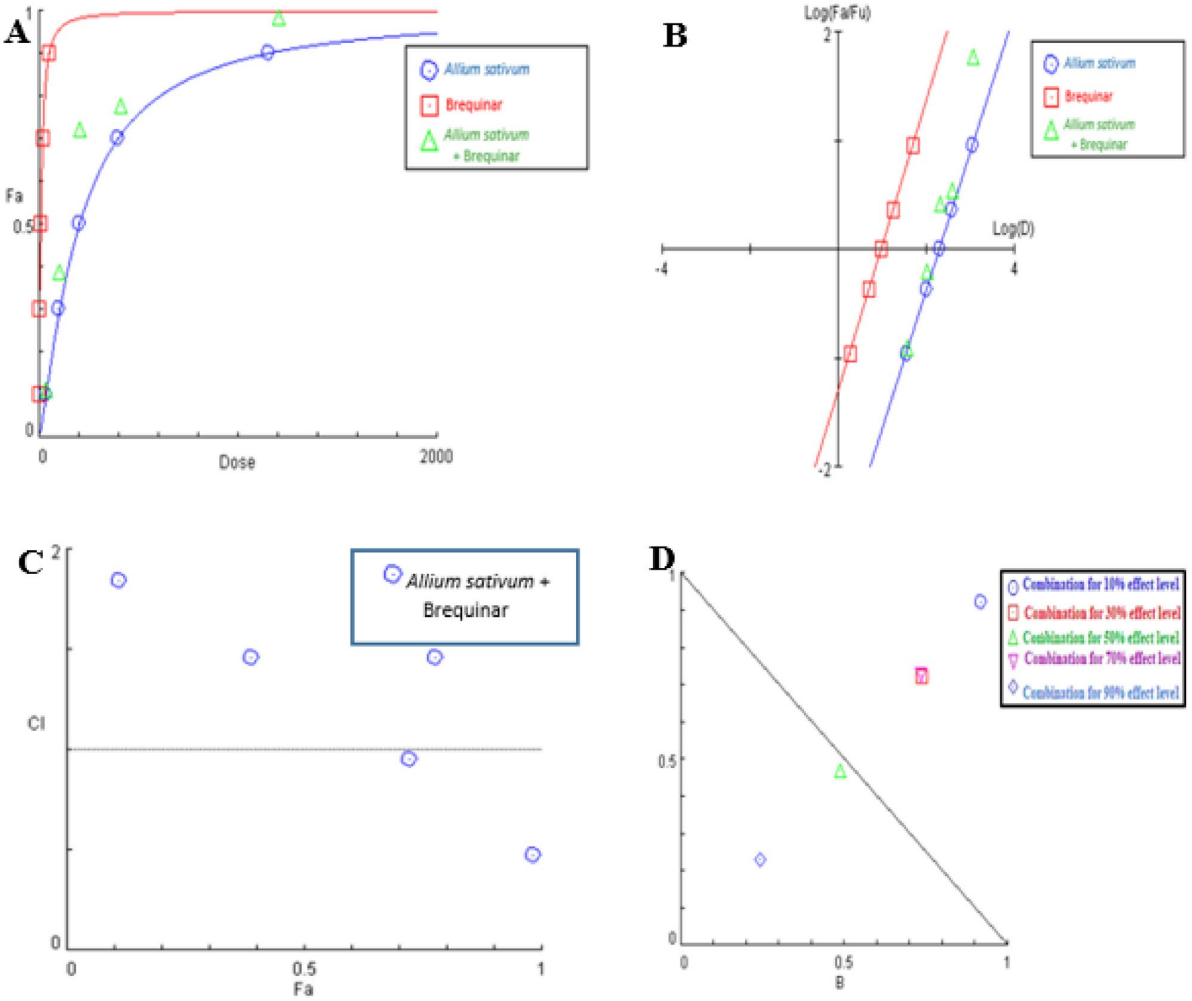
Analysis of *Allium sativum* extract and brequinar combination: (**A**) dose-effect curve and its linearization with (**B**) median-effect plot for each single drug and combination treatment (**C**) combination index plot and (**D**) dose-normalized isobologram for various effects (10%, 30%, 50%, 70 and 90%) in the combinations of *Allium sativum* extract and brequinar.

The combination of *Allium sativum* extract with brequinar markedly decreased the enzymatic activity in comparison with single component treatment. The dose-effect curve and its linearization with median-effect plot for each single drug and combination treatment were shown in Fig. 11A,B, respectively. Across the total range of drug effect levels, the interactions of *Allium sativum* extract were generally on the synergistic side at 50% and 90% effect levels (CI < 1) (Fig. 11C) showing that the hindrance of hDHODH activity was significantly increased when *Allium sativum* extract was mixed with brequinar at doses 431.44 and 5062.94 ug/mLas outlined in Table 5.

**Table 5:** Fractional inhibition of hDHODH, combination index values (CI) and DRI values of *Allium sativum* extract and brequinar in combination at several effect levels.

(Fa × 100) % (hDHODH) Inhibition of the combined drugs (%)	CI values	Dose (ug/ml) *Allium sativum* extract	Dose (nM) Brequinar	DRI *Allium sativum* extract	DRI Brequinar
11	1.84 (Ant)	39.32	2.05	1.09	1.09
39	1.46 (Ant)	142.08	6.96	1.38	1.35
72	0.95 (Syn)	431.44	19.99	2.14	2.05
78	1.46 (Ant)	545.62	24.99	1.38	1.36
98	0.47 (Syn)	5062.94	207.41	4.38	4.08

Fa signifies fraction affected. CI < 1, = 1, and > 1 indicate synergism (Syn), additive effect (Add), and antagonism (Ant), respectively. DRI > 1 indicates favourable dose reduction (in fold) for the drug in combination.

Isobolographic study, which graphically represented variations in the magnitude of interactions as a function of *Allium sativum* extract and brequinar concentrations illustrated that the combination data points were settled under the additivity line at effect levels 50% and 90% inhibition, indicating synergism (Fig. 11D). Whereas, at 10%, 30% and 70% effect levels, the combination data points were settled over the line of additivity indicating that *Allium sativum* extract doses 39.32, 142.08 and 545.62 (ug/mL) in combination with brequinar, generated a lower effect of inhibition than the predicted from their addition, implying antagonism (Fig. 11D).

Moreover, our dose reduction index (DRI) analysis (Table 5) showed that brequinar in this combination could be utilized at a dose 2.05 and 4.08-fold less when compared to its single-use to produce 72% and 98% inhibition for hDHODH enzyme, respectively.

These findings strongly suggest that the combination of *Allium sativum* extract and brequinar should be studied further in vivo and in clinical trials since it offered a stronger therapeutic impact with a better safety profile due to the lower doses required for each individual drug in the combination treatment.

#### Analysis of silibinin and brequinar binary mixture

**Figure 12:**
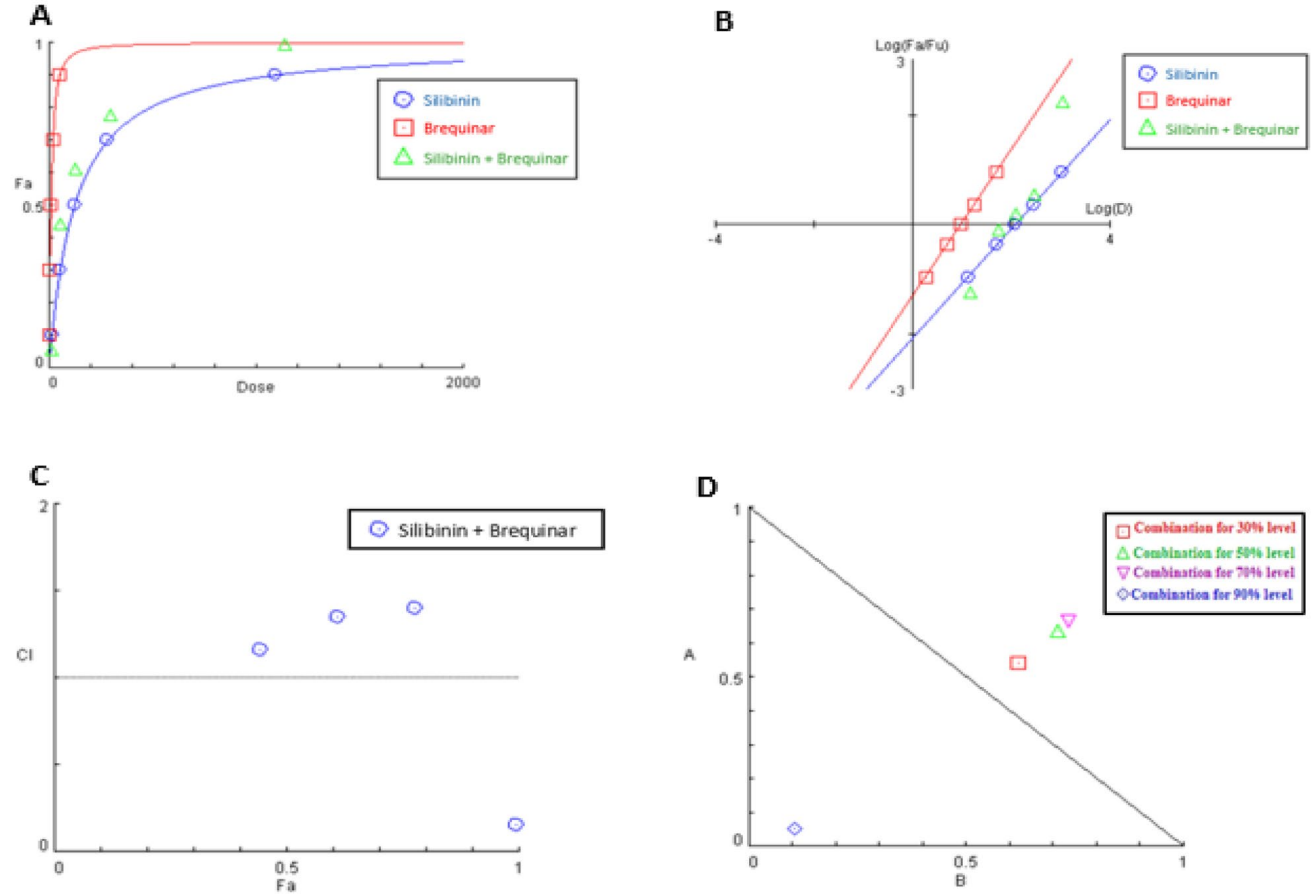
Analysis of silibinin and brequinar combination: (**A**) dose-effect curve and its linearization (**B**) median-effect plot for each single drug and combination treatment (C) combination index plot and (**D**) dose-normalized isobologram for various effects (10%, 30%, 50%, 70 and 90%) in the combinations of silibinin and brequinar.

The dose-effect curve and its linearization with median-effect plot for each single drug and combination treatment were shown in Fig. 12A,B, respectively. The outcomes appeared in Table 6 revealed that when silibinin and brequinar were combined, CI value at the 90% effect level was consistently less than 1, demonstrating synergistic collaboration and by the same way, the associated combination data point was settled under the additivity line in its isobologram as shown in Fig. 12D. Whereas the CI at 30%, 50% and 70% effect levels of inhibition demonstrated in Fig. 12C, readily illustrated moderate antagonism with combination index values of 1.14, 1.34 and 1.4, respectively (Table 6) and, as a result, their associated combination data points in the isobologram were situated above the additivity line (Fig. 12D).

**Table 6:** Fractional inhibition of hDHODH, combination index values (CI) and DRI values of silibinin and brequinar in combination at several effect levels.

(Fa × 100) % (hDHODH) Inhibition of the combined drugs (%)	CI values	Dose (nM) Silibinin	Dose (nM) Brequinar	DRI Silibinin	DRI Brequinar
6	3.49 (Ant)	7.23	1.17	0.53	0.62
44	1.14 (Ant)	97.72	8.26	1.85	1.61
61	1.34 (Ant)	191.74	13.71	1.57	1.40
78	1.40 (Ant)	426.52	24.99	1.50	1.36
99	0.16 (Syn)	21,481.50	474.52	19.63	9.33

Fa signifies fraction affected. CI < 1, = 1, and > 1 indicate synergism (Syn), additive effect (Add), and antagonism (Ant), respectively.DRI > 1 indicates favourable dose reduction (in fold) for the drug in combination.

Furthermore, DRI analysis (Table 6) revealed that in this combination, brequinar could be utilized at a dose 9.33-fold less when compared to its single-use to produce 99% inhibition for hDHODH enzyme (Table 6). These findings confirmed that this binary mixture is an excellent combination providing better therapeutic effects and lower side effects.

#### Analysis of silymarin standard extract and brequinar binary mixture

**Figure 13:**
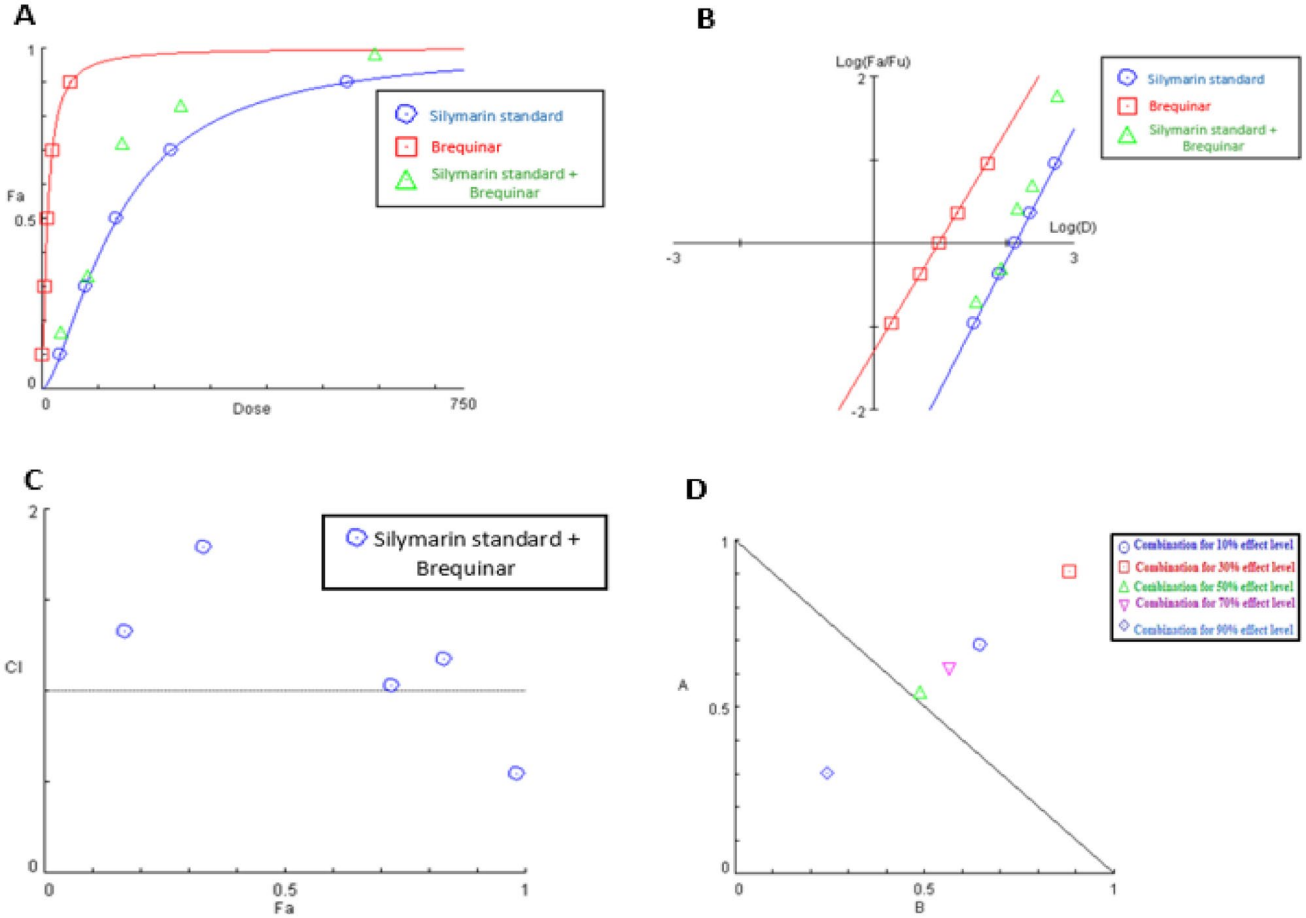
Analysis of silymarin standard and brequinar combination: (**A**) dose-effect curve and its linearization (**B**) median-effect plot for each single drug and combination treatment (**C**) combination index plot and (**D**) dose-normalized isobologram for various effects (10%, 30%, 50%, 70 and 90%) in the combinations of silymarin standard and brequinar.

In the same manner, the dose-effect curve and its linearization with median-effect plot for each single drug and combination treatment were shown in Fig. 13A,B, respectively. The results appeared in (Table 7) implied that when silymarin standard extract and brequinar were combined, the combination index value at the 90% effect level was consistently less than 1, demonstrating synergistic interaction and subsequently, the associated combination data point was settled under the additivity line in its isobologram as shown in Fig. 13D. Whereas the CI at 50% inhibition demonstrated in Fig. 13C, showed an additive collaboration and thus its data point in the isobologram was settled on the line of additivity. Meanwhile the CI values at 10%, 30% and 70% effect levels of inhibition demonstrated in Fig. 13C, readily illustrated antagonism with combination index values of 1.33, 1.79 and 1.14, respectively (Table 7) and, as a result, their associated combination data points in the isobologram were situated above the additivity line (Fig. 13D). In addition, DRI analysis (Table 7) revealed that, brequinar in this combination could be utilized at a dose 4.08-fold less when compared to its single-use to produce 98% inhibition for hDHODH enzyme (Table 7).

**Table 7:** Fractional inhibition of hDHODH, combination index values (CI) and DRI values of silymarin standard extract and brequinar in combination at several effect levels.

(Fa × 100) % (hDHODH) Inhibition of the combined drugs (%)	CI values	Dose (ug/ml) Silymarin standard extract	Dose (nM) Brequinar	DRI Silymarin standard extract	DRI Brequinar
17	1.33 (Ant)	47.76	2.92	1.46	1.55
33	1.79 (Ant)	85.75	5.81	1.10	1.13
72	1.03 (Add)	245.88	19.99	1.84	2.05
83	1.14 (Ant)	373.37	32.64	1.63	1.77
98	0.55 (Syn)	1805.11	207.41	3.32	4.08

Fa signifies fraction affected. CI < 1, = 1, and > 1 indicate synergism (Syn), additive effect (Add), and antagonism (Ant), respectively. DRI > 1 indicates favourable dose reduction (in fold) for the drug in combination.

## Experimental

### Construction of an in-house database of immunosuppressive plants

Two thousand one hundred and fifty-four phytochemical components from 32 selected immunomodulatory plants (Table S1), known traditionally to heal immune related disorders or based on previous in vitro*, *in vivo or clinical studies from literature review, were obtained for construction of an in-house database (Table S3) depending on a previous literature analysis of their chemical makeup. The two-dimensional structures of these compounds as well as the reference medication, brequinar (CID 57030), were obtained in (.sdf) format from the National Centre for Biotechnology Information's PubChem database (https://pubchem.ncbi.nlm.nih.gov/) and Dictionary of natural products (http://dnp.chemnetbase.com/faces/chemical/ChemicalSearch.xhtml;jsessionid=0A16BF52515E734B15A96DCBAE7788B9). Chemdraw software (CambridgeSoft Corporation, Cambridge, USA) was used to draw structures and to store them as (.sdf) files.

### Preparation of ligand structures

The Lig Prep 2.3 module (Lig Prep, version 2.3, 2015, Schrödinger, USA) was used to construct the 3D structure for each compound and look for alternative conformers. The OPLS force field (OPLS 3, Schrödinger, USA) was used to geometrically optimize each ligand structure and to compute the partial atomic charges. Finally, 32 poses with distinct steric characteristics for each ligand were created for subsequent docking investigations.

### ADME/Tox analysis

The ADMET (Absorption, Distribution, Metabolism, Excretion, and Toxicity) properties of lead compounds were assessed using the QikProp module of Schrodinger's Maestro 10.2 interface. Various physio-chemical characteristics were estimated to account for the investigated molecules potential as effective therapeutic candidates.

### In silico screening of immunomodulatory database for hDHODH inhibitors

For structure-based in silico screening and prediction of the binding mode of the top scoring phytochemicals-hDHODH complexes, the software package Schrodinger Maestro 10.2 (LLC, New York, NY) was employed. On the Maestro 10.2 panel interface, (Maestro, version 10.2, 2015, Schrödinger, USA), the chemical structure of each component was viewed and their interactions were investigated.

### Retrieval and preparation of target protein structure

The X-ray crystal structure of human dihydroorotate dehydrogenase (hDHODH) in complex with a leflunomide derivative inhibitor 4 (PDB ID: 3G0U) was picked and retrieved from RCSB Protein Data Bank (http://www.rcsb.org/pdb). The selection of the crystal structure (3G0U) was relied on some criteria such as it was the best resolution available (2.0 Å), co-crystallized with a leflunomide derivative inhibitor 4 and from human source.

The crystal structure of the target protein was downloaded as pdb file, then prepared and optimized by minimizing the energy utilizing the protein preparation wizard (OPLS 3 force field) module executed in Schrodinger suit. Hydrogen bonds and bond order were assigned after the protein optimization was performed. At pH 7, zero order bonds to metals and disulphide bonds were also constructed. Besides, the water molecules in hDHODH were eliminated. For grid box generation, the residues involved in the interactions with the co-crystallized ligand were utilized to build the grid.

### Molecular docking

The Glide 10.2 module (Glide, version 10.2, 2015, Schrödinger, USA) was used to dock the reduced and refined compounds from the Lig Prep file in extra-precision (XP) mode, with default settings set. The empirical scoring function of the Glide-Dock program was used to create modelling scores. 2D and 3D ligand-target protein interactions as ion pairs, hydrogen bonds and hydrophobic interactions have been demonstrated in the Maestro interface to investigate their most preferred binding modes. Subsequent to molecular docking, the sum of the fitness scores of the active hits detected among each plant determining its total in silico activity was calculated. Since The number of phyto-constituents in the constructed database varied from plant to other, so in order to compare various plants, the total in silico activity of each plant (sum of docking scores of the retrieved hits) was multiplied by the ratio of the number of active hits to the total number of constituents present in that plant in the database giving a value of (plant score) according to the following equation^41^:

1 $${\text{Plant}}\;{\text{ score }} = {\text{ Sum}}\;{\text{of}}\;{\text{docking }}\;{\text{scores}}\;{\text{of }}\;{\text{the}}\;{\text{ retrieved }}\;{\text{hits }} \times {\mkern 1mu} \left( {{\text{no}}\;{\text{ of}}\;{\text{ active}}\;{\text{ hits }}\;{\text{retrieved }}/{\text{ total}}\;{\text{ no }}\;{\text{of}}\;{\text{ plant}}\;{\text{ compounds}}\;{\text{ in }}\;{\text{database}}} \right).$$

The top five scored plants, standardized silymarin extract (purchased from Pharco Pharmaceuticals) in addition to the top-scored compounds; silybin and silychristin B (purchased from Sigma-Aldrich Chemical Co.), were promoted to further in vitro study to confirm their activities.

### Molecular dynamics simulation

Molecular dynamics (MD) simulations were performed with Gromacs 2020.1 version to examine the stability of protein–ligand complexes obtained from molecular docking studies with Glide. Necessary input files were prepared via the CHARMM -GUI server. Topology files of proteins and compounds were prepared using Charmm36m force fields. It was solvated with the TIP3 water model, using the rectangular box type 10 Å away from the protein–ligand complexes, and neutralized by adding 0.15 KCl salt. The created system was minimized to 5000 nsteps with the steep integrator. It was equilibrated by Nose–Hoover and Parrinello-Rahman algorithms with 0.5 ns duration NVT/NPT ensemble steps at 300 K and 1 atm pressure. 100 ns MD simulation of 1000 frames was run. The root mean square deviation (RMSD), The root mean square fluctuation (RMSF), and hydrogen bond (H bond) analyses were calculated with gmx scripts and binding free energy molecular mechanics poisson-boltzmann surface area (MM-PBSA) was calculated with g_mmpbsa script. RMSD, RMSF, and H bonds graphs were created with GraphPad Prism 8. MD trajectory videos and protein–ligand binding poses visualization were created with PyMol Molecular Graphics System version 2.5.2 software.

### In vitro assay of hDHODH inhibitory activity of top-scored plants and compounds

#### Preparation of the extracts/compound test solutions

The top five scored plants (*Zingiber officinale, Curcuma longa, Glycyrrhiza glabra, Allium sativum and Olea europaea*) were purchased from a well reputed local Egyptian market in Alexandria, Egypt. The sample were authenticated by Professor Sania Ahmad, Faculty of Science, Alexandria University via macroscopical and microscopical examination of the tested samples. A voucher specimens were deposited in the herbarium of Pharmacognosy Department, Faculty of Pharmacy, Alexandria University under codes of (ZO 001, CL 007, GG 004, AS 2022 and OE 008), respectively. Our study complies with relevant institutional, national, and international guidelines and legislation. The plants were air dried and separately extracted by double maceration on 70% ethanol. The extracts were then filtered and evaporated under reduced pressure using a rotary evaporator (BuchiRotavapor Model R-200, Flawil, Switzerland). Each tested dry plant extract (1 mg) together with silymarin standard, silibinin and silychristin B was separately placed in a 10 mL volumetric flask, dissolved in 1 mL dimethyl sulfoxide (DMSO) and the volume was adjusted to 10 mL with distilled water to prepare the stock solution for each tested sample. Stock solutions of each examined sample were finally diluted utilizing the assay buffer solution (100 mM Tris and 0.1% Triton X-100, pH 8.0) to create various concentrations of each sample.

#### In vitro assay of hDHODH inhibitory activity

The 2,6-dichlorophenolindophenol (DCIP) colorimetric test was used to estimate the hDHODH activity, as portrayed by Copeland et al.^42^. This is a coupled examination method in which oxidation of dihydroorotate and ensuing reduction of coenzyme Q6 are stoichiometrically comparable to the reduction of DCIP. The drop in absorbance at 600 nm is linked to a reduction in DCIP.

The assay mixture is composed of 25 nM hDHODH, 135 μM Co-enzyme Q6, (100 mM Tris and 0.1% Triton X-100, pH 8.0) and 50 μM DCIP and was prepared just before use. Incubation of 180 μL solution from hDHODH enzyme in assay buffer with 10 μL of different concentrations of extract solutions for 10 min was performed. The reaction was started by the addition of 10 μL of dihydroorotate for a final concentration of 500 μM and reporting the decline in absorbance at 600 nm for 5 min during which the velocity stayed straight at one-minute intervals on a microplate reader. Velocities are accounted for as the variation in absorbance at 600 nm per minute (∆ A/min).

For each tested sample, the data was plotted as optical density (OD) versus time. The range of time points during which the reaction is linear was determined and then the reaction velocity (V) was obtained in OD/min. Finally, the slope of a line fit to the linear part of the data plot was determined. Wells without inhibitors serve as the highest DCIP reduction point (highest hDHODH activity) and brequinar (purchased from Sigma-Aldrich Chemical Co.) was employed as a positive control. The activity was measured as percent inhibition of hDHODH enzyme. Inhibition (%) was calculated according to the accompanying equation:

2 $${\text{Inhibition }}\left( \% \right) \, = \, \left( {{\text{slope of EC}} - {\text{slope of S}}} \right)/ \, \left( {\text{slope of EC}} \right){\text{ X1}}00$$

where: Slope of EC is the slope of enzyme control and slope of S is the slope of the sample screened. The IC_50_ values (concentrations inducing half-maximal inhibition) for the tested extracts were calculated by fitting the experimental data to a dose-response nonlinear regression curve utilizing Graphpad Prism (Version 6.01) software.

### The study of synergistic inhibitory activity on hDHODH enzyme

#### The combination effects determined using the ‘‘fixed ratio’’ method

The plant with highest in vitro activity; *Allium sativum* (the most reduced IC_50_), in addition to silymarin standard and silibinin were exposed to combination analysis on hDHODH enzyme with brequinar as a reference, the generally utilized immunosuppressive medication. The hDHODH inhibitory action of the three combinations was examined with the technique depicted under (“In vitro assay of hDHODH inhibitory activity” section). The doses of tested samples and brequinar that displayed an effect level of enzymatic activity decline (10%, 30%, 50%, 70% and 90%) were redefined and summed up in Table 8.

**Table 8:** Illustrative table demonstrating the tested data points of *Allium sativum,* silymarin standard extract and silibinin samples conducted in combination analysis at five dose levels.

Effect level (EC_x_)	Dose (nM) Brequinar	Dose (ug/ml) *Allium sativum* extract	Dose (ug/ml) Silymarin standard extract	Dose (nM) Silibinin
EC_10_	3.78	72.42	65.58	27.12
EC_30_	10.28	205.82	155.38	105.84
EC_50_	19.54	403.56	267.26	243.76
EC_70_	36.82	791.9	459.28	567.62
EC_90_	101.74	2314.24	1086.42	2189.06

EC_10_, EC_30,_ EC_50_, EC_70_ and EC_90_, are the concentrations of each individual sample required to inhibit hDHODH enzyme at 10, 30, 50, 70 and 90%, respectively.

The immunosuppressive effects of the three combinations on hDHODH enzyme for estimation of the drug-drug combination nature were deeply studied using the median- effect, combination index, isobolographic, and dose reduction index analyses.

### The median- effect analysis approach

The dose-effect sigmoidal curves for each individual active ingredient and its binary combination was simply plotted using the easily operated CompuSyn software (Chou and Martin, 2005, Compusyn Inc, USA), and then converted into the linear median-effect plot using the median-effect equation proceeded from the general mass-action low principle. This principle established a reasonable connection between a single issue and multiple issues^39,43^. The following is a breakdown of the median-effect equation (MEE)^44^:

3 $$\frac{fa}{{fu}} = \left( \frac{D}{Dm} \right)^{m}$$

Equation (3) can be reconstructed into:

3.1 $$log\left( {\frac{fa}{{fu}}} \right) = m\log \left( D \right) - m {\text{log}}\left( {Dm} \right)$$

where D is the dose of a drug, fa is the fraction influenced by D (i.e. percentage effect/100), Dm is the median-effect dose (equivalent to IC_50_ or ED_50_) that decreases the enzyme activity by 50%, *fu* is the unaffected fraction (*fu* = *1-fa*) and finally m is the slope of the median-effect plot. In the median-effect plot, y = log (*fa/fu*) versus x = log (D), log (Dm) is the x-intercept. The suitability of the data to the principle of mass action is revealed by the linear correlation coefficient (r) of the median effect plot, which is generally > 0.97 in in vitro assays^45^.

### Isobolographic analysis

Isobologram plot is composed of two-axes, in which these axes represent medication A and B concentrations, respectively. The required doses to produce a specific effect x from each separate medication A and B (for example, IC_50_ of drug A and IC_50_ of drug B when x = 50 percent) were plotted on the x and y coordinates, respectively. Connecting these two points (e.g., (IC_50_ of A, 0) and (0, IC_50_ of B) for a 50% effect) yielded the diagonal line of additivity. Antagonism, additivity, and synergism were denoted by data points above, on, and below the additivity line, respectively^46^.

Isobolograms are used to demonstrate dose-dependent interactions of concomitant medications at different levels of efficacy^47^. The combined effect could be less, equal, or greater than expected from the individual effects.

### Combination index analysis

When the median-effect equation is merged with isobologram-combination index equation (CI), a quantitative measurement of the concomitant activities of drug combinations at various effect levels is obtained. The following formula was used to automatically calculate the CI numerical values^39,45^:

4 $${\text{CI}} = \frac{{{\text{Da}}}}{{\left( {{\text{Dx}}} \right){\text{a}}}} + \frac{{{\text{Db}}}}{{\left( {{\text{Dx}}} \right){\text{b}}}}$$

where (Dx)a and (Dx)b are the doses of the individual compounds required to reduce the enzyme activity at the x-effect level, while Da and Db are the combined compound doses that provide the same effect. The CI is used to determine if the combination is synergistic (CI < 1), additive (CI = 1), or antagonistic (CI > 1). Furthermore, by plotting combination indices against a series of effect levels, the combination index (*fa*-CI) plot could be automatically generated using the compusyn software.

Note that the effect-oriented plot *(fa*-CI) and the dose-oriented isobologram are two aspects of the same thing; both are based on the median-effect equation and hence yield the same result of synergism, addition or antagonism.

### Dose reduction index (DRI) analysis

The Dose Reduction Index (DRI) of a combination of two drugs reveals how many times each drug in a synergistic combination achieves a dose reduction at a specific effect level (*f*_*a*_)^47^. In clinical practice, reducing the dose while maintaining the same therapeutic effect leads to a decrease in the potential toxicity profile to the host. (DRI) was calculated automatically using the following formula on the computer software CompuSyn^39^:

5 $${\text{DRI }} = \frac{{\text{EDX of compound alone }}}{{ {\text{EDX of compound in combination with combination partner}} }}$$

The Dose reduction index > 1 is beneficial since it indicates a reduction in the doses of the concomitant drugs while maintaining the same efficacy.

### Statistical analysis

Statistical analysis was carried out using the Excel (Microsoft, 2016), Graphpad Prism software and Computer program CompuSyn.

## Conclusion

In conclusion, the so obtained hits specifically silibinin, in pursuit of virtual screening of an in-house data base of naturally occurring immunomodulatory phytoconstituents; were found to be promising hDHODH inhibitors. Molecular docking and MD simulations revealed possible binding modes inside the hDHODH active site, which helped to explain the potential for silibinin and brequinar on hDHODH catalysis. These in silico results were further confirmed via in vitro assay. Furthermore, combination analysis by fixed ratio design revealed that combining each of *Allium sativum*, silymarin and silibinin with brequinar suppressed hDHODH activity synergistically, resulting in a favorable dose reduction of synthetic medicines. However, more in vivo investigations are needed to confirm the proposed combination superior activity and to reveal its overall therapeutic mechanism in immunosuppression.

## Supplementary Information

Supplementary Information 1.

Supplementary Information 2.

Supplementary Information 3.

Supplementary Information 4.

## Data Availability

All data generated or analyzed during this study are included in this article (and its supplementary information files).
