# Supplementary material for: Discovery of potential natural dihydroorotate dehydrogenase inhibitors and their synergism with brequinar via integrated molecular docking, dynamic simulations and in vitro approach
Source: Sci Rep. 2022 Nov 9;12:19037. doi: 10.1038/s41598-022-23006-1 (PMC9646789; doi:10.1038/s41598-022-23006-1)
Supplement: Supplementary file 3 — Supplementary Information 3. [file 41598_2022_23006_MOESM3_ESM.docx]

| 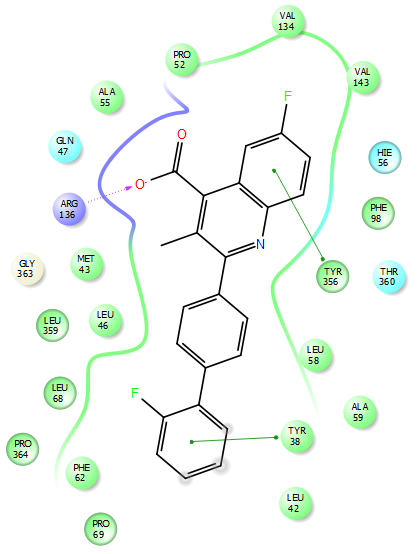  **A** | 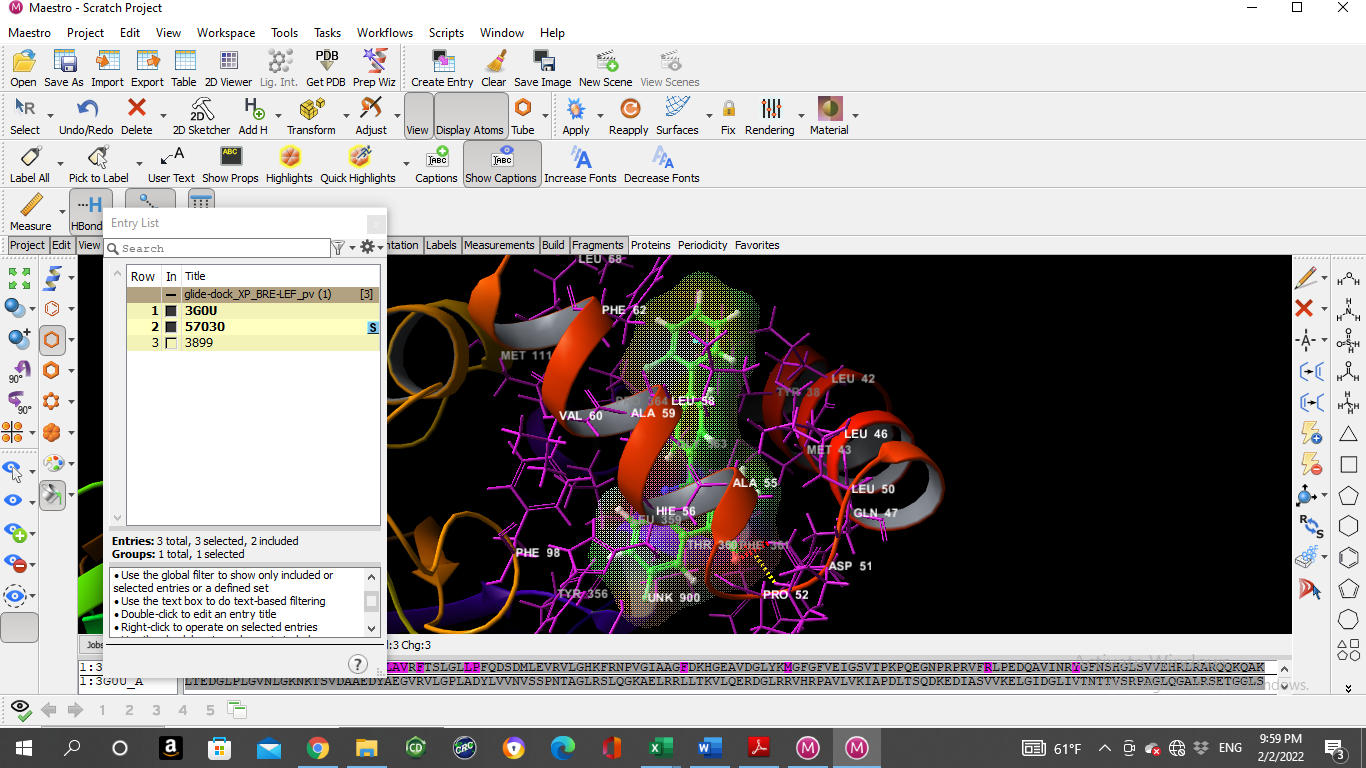  **B** |
| --- | --- |

**Figure S1.** The docked pose of brequinar interacting with the amino acid residues of hDHODH active site in 2D (A) and 3D (B) sketches.


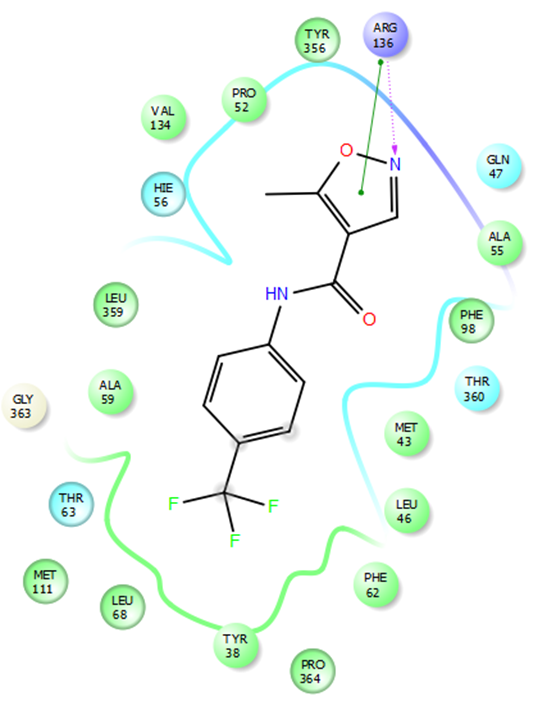


**B**

**A**


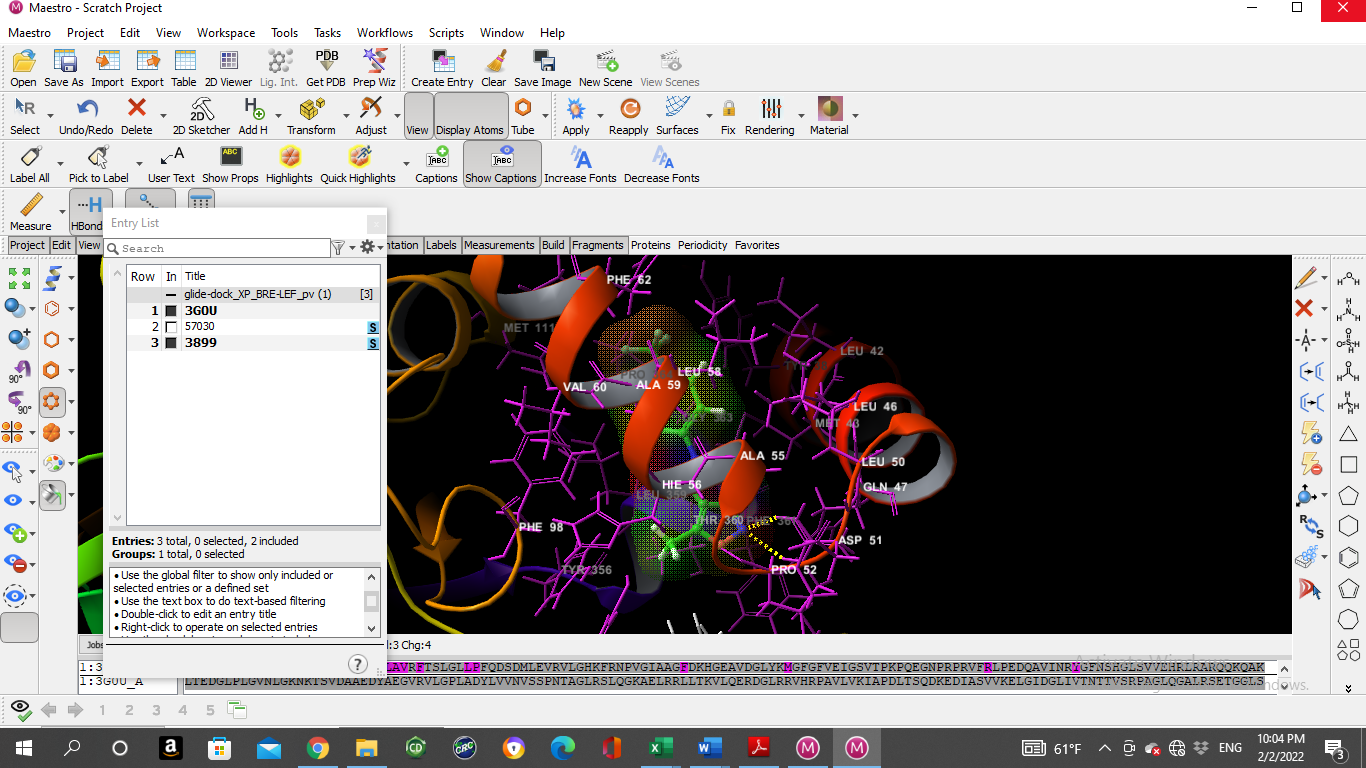


**Figure S2.** The docked pose of leflunomide (co-crystallized ligand) interacting with the amino acid residues of hDHODH active site in 2D (A) and 3D (B) sketches.

**Table S1: An in-house database of 2154 compounds from 32 immunosuppressive plants (see attached supplementary excel file).**

**Table S2: Results of ADME and drug-likeness analysis of the constructed database (see attached supplementary excel file).**

**Table S3. The selected 32 immunomodulatory plants known traditionally to heal immune related disorders or based on their previous *in vitro* or *in vivo* studies from literature review.**

| **Plant name** | **Family** | **Pharmacological action** | **Reference** |
| --- | --- | --- | --- |
| ***Allium sativum*** | Amaryllidaceae | *In vitro:* alliin was able to suppress LPS inflammatory signals - prevent the pro-inflammatory cytokines IL-6 and MCP-1. | [^1^](#_ENREF_1) |
| ***Artemisia annua*** | Asteraceae | Traditionally: used for autoimmune diseases such as systemic lupus erythematosus and rheumatoid arthritis.  *In vivo*: suppresses delayed type hypersensitivity reaction.  *In vitro*: had inhibitory effect on calmodulin. | [^2-4^](#_ENREF_2) |
| ***Curcuma longa*** | Zingiberaceae | Traditionally: anti-inflammatory agent.  *In vitro*: curcumin was found to have potent suppressive effects on the production of several pro-inflammatory mediators, including TNF*α*, IL-1*β*, IL-6, IL-12, and IL-8, it also suppresses TH1 and TH17 -mediated inflammatory responses.  *In vivo*: reduced the number of macrophage in atherosclerotic lesions. | [^5-9^](#_ENREF_5) |
| ***Cichorium intybus*** | Asteraceae | Traditionally: anti-inflammatory, anti-rheumatic, anti-arteriosclerotic and antiarthritic.  *In vitro:* showed an inhibitory effect on lymphocyte proliferation assay in the presence of PHA (phytohemagglutinin). | [^10-13^](#_ENREF_10) |
| ***Calendula officinalis*** | Asteraceae | Traditionally: anti-inflammatory, analgesic and anti-tumoral.  *In vitro:* inhibited the mitogen-induced lymphocyte proliferation, anti-inflammatory activities. | [^10^](#_ENREF_10)^,^ [^14^](#_ENREF_14) |
| ***Cannabis sativa*** | Cannabaceae | Traditionally: used to treat rheumatic pains.  *In vitro:* suppressed the hyperactivity of immune system by acting on cannabinoid receptors such as cannabinoid receptor 1 (CB1) and cannabinoid receptor 2 (CB2).  Also, exerted a direct effect on immune cells by inhibiting the proliferation of lymphocytes. | [^1^](#_ENREF_1)^,^ [^15^](#_ENREF_15)^,^ [^16^](#_ENREF_16) |
| ***Camellia sinensis*** | Theaceae | Traditionally: used for treatment of autoimmune arthritis.  *In vitro*: the polyphenolic compound (catechin) present in green tea possessed anti-inflammatory properties. | [^1^](#_ENREF_1)^,^ [^17^](#_ENREF_17) |
| ***Citrullus colocynthis*** | Cucurbitaceae | Traditionally: used as remedies against cancer, inflammation, anti-rheumatic, and anti-arthritic.  *In vitro:* reduced the cell proliferation induced by concanavalin A (con-A). | [^14^](#_ENREF_14)^,^ [^18^](#_ENREF_18)^,^ [^19^](#_ENREF_19) |
| ***Citrus aurantium*** | Rutaceae | Traditionally: used to treat the inflammation  *In vitro:* active against concanavalin A induced proliferation in thymocytes, also active against LPS (lipopolysaccharide)-induced proliferation in splenocytes. | [^20^](#_ENREF_20)^,^ [^21^](#_ENREF_21) |
| ***Cynara scolymus*** | Asteraceae | *In vitro*: cynarin was used as an immune‐suppressive agent.  *In vivo:* caused an increase in the basal activity of reactive oxygen species from macrophages and the highest dose of *C. scolymus* was used for suppression of the delayed-type hypersensitivity response. | [^22^](#_ENREF_22)^,^ [^23^](#_ENREF_23) |
| ***Euphorbia species***  ***(E. royleana***  ***E. lactea***  ***E. tirucalli***  **Boiss)** | Euphorbiaceae | Traditionally: anti-allergic.  *In vitro:* inhibited delayed-type hypersensitivity reaction, inhibited antibody synthesis, reduced CD4+ T cells and neutrophils, inhibited the process of phagocytosis and showed inhibition of intracellular interleukin-2. | [^2^](#_ENREF_2)^,^ [^11^](#_ENREF_11)^,^ [^24^](#_ENREF_24)^,^ [^25^](#_ENREF_25) |
| ***Ephedra sinica*** | Ephedraceae | Traditionally: anti-inflammatory.  *In vitro:* the polysaccharide fractions (A, B, C) acquired from the stems showed an inhibitory effect on the splenocyte proliferation.  *In vivo*: possessed an inhibitory action of the polysaccharide B fraction (PB) on humoral immune response. | [^26^](#_ENREF_26)^,^ [^27^](#_ENREF_27) |
| ***Glycyrrhiza glabra*** | Fabaceae | Traditionally: relief of inflammation and rheumatitis.  *In vitro*: suppressed allergic reactions, down-regulated IgE production, inhibited the classical pathway of complement system, inhibited calcineurin activity and T cell proliferation. | [^2^](#_ENREF_2)^,^ [^28^](#_ENREF_28)^,^ [^29^](#_ENREF_29) |
| ***Lawsonia inermis*** | Lythraceae | Traditionally: treatment of burns, edema and healing wounds  *In vitro:* inhibited lymphocyte transformation. | [^2^](#_ENREF_2)^,^ [^30^](#_ENREF_30) |
| ***Linum usitatissimum*** | Linaceae | Traditionally: immunomodulatory, anti-inflammatory and anti-tumor.  *In vitro*: showed inhibitory effect on mitogen (concanavalin A)-induced response of human peripheral blood lymphocytes, which was comparable to that of cyclosporin A. | [^31^](#_ENREF_31)^,^ [^32^](#_ENREF_32) |
| ***Matricaria chamomilla*** | Asteraceae | Traditionally: possessed anti-inflammatory effect.  *In vitro*: disrupting Th1/Th2 balance to Th1 upregulation and had the ability for reducing IL-6 and TNF*α* production. | [^33-35^](#_ENREF_33) |
| ***Nigella sativa*** | Ranunculaceae | Traditionally: anti-tumour, anti-inflammatory.  *In vivo*: *Nigella sativa* volatile oil acted as a potential agent in reducing serum antibody titre, decreased splenocytes and neutrophils, dysregulated intracellular killing, cytokine production and phagocytosis. | [^36-38^](#_ENREF_36) |
| ***Oleu europea*** | Oleaceae | Traditionally: anti-inflammatory  *In vitro*: modulated the production of pro-inflammatory mediators, reduced the expression of COX2 at both the mRNA and protein level and caused a clear dose-dependent reduction of PGE2. | [^39^](#_ENREF_39)^,^ [^40^](#_ENREF_40) |
| ***Punica granatum*** | Lythraceae | Traditionally: treatment of inflammation.  *In vitro:* inhibited activation of the nuclear factor of activated T cells.  *In vivo*: decreased CD3+ T cell infiltration of the inflamed tissue. | [^2^](#_ENREF_2)^,^ [^41^](#_ENREF_41) |
| ***Propolis*** | Bee product | Traditionally: anti-inflammatory and antioxidant.  *In vitro*: at high concentrations had inhibitory effects on lymphocyte proliferation which might be linked to its anti-inflammatory properties. However, at low concentrations the effect was reversed, causing stimulation of lymphocyte proliferation. | [^42^](#_ENREF_42)^,^ [^43^](#_ENREF_43) |
| ***Panax ginseng*** | Araliaceae | Traditionally: used for immunological diseases  *In vivo*: inhibited helper T cell 17 (Th17) differentiation and promoted regulatory T (Treg) cell differentiation under Th17-polarizing condition. In addition, ginsenoside Rg3-fortified Korean red ginseng extract could suppress helper T cells, decreased Th1 cytokines and decreased pro-inflammatory cytokines. | [^44-46^](#_ENREF_44) |
| ***Piper cubeba*** | Piperaceae | Traditionally: treatment of rheumatic and inflammatory disorders.  *In vitro*: reduced the cell proliferation induced by concanavalin A. | [^18^](#_ENREF_18)^,^ [^47^](#_ENREF_47) |
| ***Physalis peruviana*** | Solanaceae | Traditionally: used to treat rheumatism.  *In vitro*: reduced the release of interleukin-6, interleukin-8, and MCP-1 in a dose-dependent manner. | [^48^](#_ENREF_48)^,^ [^49^](#_ENREF_49) |
| ***Silybum marianum*** | Asteraceae | Traditionally: exhibited anti-carcinogenic, immunomodulatory and anti-inflammatory activities.  *In vitro:* showed strong inhibitory activity on phytohaemagglutinin (PHA)-induced lymphocyte proliferation at low concentrations, inhibitory effect on blastogenesis induced by lectins and by anti-CD3 monoclonal antibody at lower concentrations. | [^10^](#_ENREF_10)^,^ [^50^](#_ENREF_50) |
| ***Salvia officinalis L*** | Lamiaceae | Traditionally: treatment of microbial infections, cancer and inflammation.  *In vitro*: used as an immunosuppressive agent through its proapoptotic effects evaluated in three human melanoma cell lines, A375, M14, and A2058. | [^51-53^](#_ENREF_51) |
| ***Sesamum indicum*** | Pedaliaceae | Traditionally: analgesic, antioxidant and anticancer.  *In vivo*: showed significant decrease in white blood cell count, erythrocyte sedimentation rate, interleukin-6 and tumor necrosis factor-*a*. | [^54^](#_ENREF_54)^,^ [^55^](#_ENREF_55) |
| ***Trigonella foenum-graecum*** | Fabaceae | Traditionally: treatment of arthritis, inflammation.  *In vitro:* inhibited growth of HL-60 myeloid cells | [^56-58^](#_ENREF_56) |
| ***Tanacetum parthenium*** | Asteraceae | Traditionally: used as a folk remedy for rheumatoid arthritis and fever.  *In vitro:* had the capacity to inhibit several pro-inflammatory enzymes including 5-lipoxygenase, phosphodiesterase-3 and phosphodiesterase-4 - It also inhibited the release of pro-inflammatory mediators nitric oxide, prostaglandin (PG) E2 and TNF*α* from macrophages and IL-4 from human peripheral blood mononuclear cells. | [^59^](#_ENREF_59)^,^ [^60^](#_ENREF_60) |
| ***Urtica dioica*** | Urticaceae | Traditionally: an infusion of the plant was used for rheumatism.  *In vitro*: reduced the cell proliferation induced by concanavalin A, showed suppressive effect on the maturation of human myeloid dendritic cells leading to reduced induction of primary T cell responses. | [^18^](#_ENREF_18)^,^ [^61^](#_ENREF_61)^,^ [^62^](#_ENREF_62) |
| ***Withania somnifera*** | Solanaceae | Traditionally: was widely used in the treatment of psoriasis, arthritis and rheumatism.  *In vitro*: produced immunosuppressive action on B and T cell activity in hyper-immune states, so that, it was widely used in the treatment of psoriasis, arthritis and rheumatism. | [^1^](#_ENREF_1)^,^ [^63^](#_ENREF_63) |
| ***Xanthium strumarium L.*** | Asteraceae | Traditionally: used as an anti-inflammatory and had potential anti-arthritic effects in the treatment of rheumatoid arthritis.  *In vivo*: suppressed paw swelling and arthritic score, increased body weight loss and decreased the thymus index, remarkably suppressed the overproduction of TNF*α* and IL-1*β* in the serum and also decreased the level of COX-2 and 5-LOX. | [^64^](#_ENREF_64)^,^ [^65^](#_ENREF_65) |
| ***Zingiber officinale*** | Zingiberaceae | Traditionally: used to decrease pain in arthritis.  *In vitro:* responsible to reduce inflammation in arthritis by inhibiting cyclooxygenase (COX) and lipoxygenase (LOX) pathways. | [^1^](#_ENREF_1)^,^ [^66^](#_ENREF_66) |

**References:**

1. Sahoo B, Banik B. Medicinal plants: source for immunosuppressive agents. Immunol Curr Res. 2018;2(106):2.

2. Amirghofran Z. Medicinal plants as immunosuppressive agents in traditional Iranian medicine. Iran J Immunol. 2010;7(2):65-73.

3. Hou L, Huang H. Immune suppressive properties of artemisinin family drugs. Pharmacology & therapeutics. 2016;166:123-7.

4. Noori S, Naderi G-A, Hassan ZM, Habibi Z, Bathaie SZ, Hashemi SMM. Immunosuppressive activity of a molecule isolated from Artemisia annua on DTH responses compared with cyclosporin A. International immunopharmacology. 2004;4(10-11):1301-6.

5. Mohammadian Haftcheshmeh S, Momtazi-Borojeni AA. Immunomodulatory therapeutic effects of curcumin in rheumatoid arthritis. Autoimmunity Reviews. 2020;19(8).

6. Jurenka JS. Anti-inflammatory properties of curcumin, a major constituent of Curcuma longa: a review of preclinical and clinical research. Alternative medicine review. 2009;14(2).

7. Abe Y, Hashimoto S, Horie T. Curcumin inhibition of inflammatory cytokine production by human peripheral blood monocytes and alveolar macrophages. Pharmacological research. 1999;39(1):41-7.

8. Hsu HY, Chu LC, Hua KF, Chao LK. Heme oxygenase‐1 mediates the anti‐inflammatory effect of Curcumin within LPS‐stimulated human monocytes. Journal of cellular physiology. 2008;215(3):603-12.

9. Coban D, Milenkovic D, Chanet A, Khallou‐Laschet J, Sabbe L, Palagani A, et al. Dietary curcumin inhibits atherosclerosis by affecting the expression of genes involved in leukocyte adhesion and transendothelial migration. Molecular nutrition & food research. 2012;56(8):1270-81.

10. Amirghofran Z, Azadbakht M, Karimi MH. Evaluation of the immunomodulatory effects of five herbal plants. Journal of Ethnopharmacology. 2000;72(1-2):167-72.

11. Khan MY, Mahadik K, Pathak AK, Namdeo A, Fulzele D. Immunomodulatory plants: a phytopharmacological review. Pharmacognosy Reviews. 2007;1(2).

12. Jarić S, Popović Z, Mačukanović-Jocić M, Djurdjević L, Mijatović M, Karadžić B, et al. An ethnobotanical study on the usage of wild medicinal herbs from Kopaonik Mountain (Central Serbia). Journal of ethnopharmacology. 2007;111(1):160-75.

13. Pushparaj P, Low H, Manikandan J, Tan B, Tan C. Anti-diabetic effects of Cichorium intybus in streptozotocin-induced diabetic rats. Journal of ethnopharmacology. 2007;111(2):430-4.

14. Duke JA. Handbook of medicinal herbs: CRC press; 2002.

15. Hegde VL, Nagarkatti M, Nagarkatti PS. Cannabinoid receptor activation leads to massive mobilization of myeloid‐derived suppressor cells with potent immunosuppressive properties. European journal of immunology. 2010;40(12):3358-71.

16. Anand U, Jacobo-Herrera N, Altemimi A, Lakhssassi N. A comprehensive review on medicinal plants as antimicrobial therapeutics: potential avenues of biocompatible drug discovery. Metabolites. 2019;9(11):258.

17. Lorenzo JM, Munekata PES. Phenolic compounds of green tea: Health benefits and technological application in food. Asian Pacific Journal of Tropical Biomedicine. 2016;6(8):709-19.

18. Daoudi A, Aarab L, Abdel-Sattar E. Screening of immunomodulatory activity of total and protein extracts of some Moroccan medicinal plants. Toxicology and industrial health. 2013;29(3):245-53.

19. Trease A. Trease and Evans Pharmacology. London. Bailliere Tindal; 1989.

20. Karthikeyan V, Karthikeyan J. Citrus aurantium (bitter orange): A review of its traditional uses, phytochemistry and pharmacology. International journal of drug discovery and herbal research. 2014;4(4):766-72.

21. Eun J-S, Yum J-Y. Effect of Aurantii nobilis Pericarpium and Aurantii immaturi Pericarpium on lmmunocytes in Mice. Korean Journal of Pharmacognosy. 1998;29(3):173-8.

22. Hueza IM, Gotardo AT, da Silva Mattos MI, Górniak SL. Immunomodulatory effect of Cynara scolymus (artichoke) in rats. Phytotherapy Research. 2019;33(1):167-73.

23. Dong G-C, Chuang P-H, Chang K-c, Jan P-s, Hwang P-I, Wu H-B, et al. Blocking effect of an immuno-suppressive agent, cynarin, on CD28 of T-cell receptor. Pharmaceutical research. 2009;26(2):375-81.

24. Kim M-S, Park S-B, Suk K, Kim IK, Kim S-Y, Kim J-A, et al. Gallotannin isolated from euphorbia species, 1, 2, 6-Tri-O-galloyl-β-D-allose, decreases nitric oxide production through inhibition of nuclear factor-κ> B and downstream inducible nitric oxide synthase expression in macrophages. Biological and Pharmaceutical Bulletin. 2009;32(6):1053-6.

25. Fernandez-Arche A, Saenz M, Arroyo M, De la Puerta R, Garcia M. Topical anti-inflammatory effect of tirucallol, a triterpene isolated from Euphorbia lactea latex. Phytomedicine. 2010;17(2):146-8.

26. Kuang H, Xia Y, Yang B, Wang Q, Wang Y. Screening and comparison of the immunosuppressive activities of polysaccharides from the stems of Ephedra sinica Stapf. Carbohydrate Polymers. 2011;83(2):787-95.

27. Trujillo WA, Sorenson WR, D CGDLJLGMRPRRCKSSCSES. Determination of ephedrine alkaloids in human urine and plasma by liquid chromatography/tandem mass spectrometry: collaborative study. Journal of AOAC International. 2003;86(4):643-56.

28. Shin Y-W, Bae E-A, Lee B, Lee SH, Kim JA, Kim Y-S, et al. In vitro and in vivo antiallergic effects of Glycyrrhiza glabra and its components. Planta medica. 2007;73(03):257-61.

29. Kroes B, Beukelman C, Van Den Berg A, Wolbink G, Van Dijk H, Labadie R. Inhibition of human complement by β‐glycyrrhetinic acid. Immunology. 1997;90(1):115-20.

30. Mikhaeil BR, Badria FA, Maatooq GT, Amer MM. Antioxidant and immunomodulatory constituents of henna leaves. Zeitschrift für Naturforschung C. 2004;59(7-8):468-76.

31. Morita H, Shishido A, Matsumoto T, Takeya K, Itokawa H, Hirano T, et al. A new immunosuppressive cyclic nonapeptide, cyclolinopeptide B from Linum usitatissimum. Bioorganic & Medicinal Chemistry Letters. 1997;7(10):1269-72.

32. Ansari R, Zarshenas MM, Dadbakhsh AH. A Review on Pharmacological and Clinical Aspects of Linum usitatissimum L. Current drug discovery technologies. 2019;16(2):148-58.

33. Asadi Z, Ghazanfari T, Hatami H. Anti-inflammatory Effects of Matricaria chamomilla Extracts on BALB/c Mice Macrophages and Lymphocytes. Iranian Journal of Allergy, Asthma and Immunology. 2020.

34. Drummond EM, Harbourne N, Marete E, Martyn D, Jacquier J, O'Riordan D, et al. Inhibition of proinflammatory biomarkers in THP1 macrophages by polyphenols derived from chamomile, meadowsweet and willow bark. Phytotherapy Research. 2013;27(4):588-94.

35. Newall CA, Anderson LA, Phillipson JD. Herbal medicines: a guide for health-care professionals: Pharmaceutical press London; 1996.

36. Nazrul Islam S, Begum P, Ahsan T, Huque S, Ahsan M. Immunosuppressive and cytotoxic properties of Nigella sativa. Phytotherapy Research: An International Journal Devoted to Pharmacological and Toxicological Evaluation of Natural Product Derivatives. 2004;18(5):395-8.

37. Salomi N, Nair S, Jayawardhanan K, Varghese C, Panikkar K. Antitumour principles from Nigella sativa seeds. Cancer letters. 1992;63(1):41-6.

38. Mutabagani A, El-Mahdy SA. A study of the anti-inflammatory activity of Nigella sativa L. and thymoquinone in rats. Saudi Pharmaceutical Journal. 1997;5:110-3.

39. Rosignoli P, Fuccelli R, Fabiani R, Servili M, Morozzi G. Effect of olive oil phenols on the production of inflammatory mediators in freshly isolated human monocytes. The Journal of nutritional biochemistry. 2013;24(8):1513-9.

40. Celano M, Maggisano V, Lepore SM, Russo D, Bulotta S. Secoiridoids of olive and derivatives as potential coadjuvant drugs in cancer: A critical analysis of experimental studies. Pharmacological Research. 2019;142:77-86.

41. Lee S-I, Kim B-S, Kim K-S, Lee S, Shin K-S, Lim J-S. Immune-suppressive activity of punicalagin via inhibition of NFAT activation. Biochemical and biophysical research communications. 2008;371(4):799-803.

42. Wolska K, Gorska A, Antosik K, Lugowska K. Immunomodulatory effects of propolis and its components on basic immune cell functions. Indian Journal of Pharmaceutical Sciences. 2019;81(4):575-88.

43. Burdock G. Review of the biological properties and toxicity of bee propolis (propolis). Food and Chemical toxicology. 1998;36(4):347-63.

44. Lee J-I, Park KS, Cho I-H. Panax ginseng: a candidate herbal medicine for autoimmune disease. Journal of ginseng research. 2019;43(3):342-8.

45. Hwang I, Ahn G, Park E, Ha D, Song J-Y, Jee Y. An acidic polysaccharide of Panax ginseng ameliorates experimental autoimmune encephalomyelitis and induces regulatory T cells. Immunology letters. 2011;138(2):169-78.

46. Song J-H, Kim K-J, Choi S-Y, Koh E-J, Park J, Lee B-Y. Korean ginseng extract ameliorates abnormal immune response through the regulation of inflammatory constituents in Sprague Dawley rat subjected to environmental heat stress. Journal of ginseng research. 2019;43(2):252-60.

47. Choi E-M, Hwang J-K. Investigations of anti-inflammatory and antinociceptive activities of Piper cubeba, Physalis angulata and Rosa hybrida. Journal of Ethnopharmacology. 2003;89(1):171-5.

48. Mier-Giraldo H, Díaz-Barrera LE, Delgado-Murcia LG, Valero-Valdivieso MF, Cáez-Ramírez G. Cytotoxic and immunomodulatory potential activity of Physalis peruviana fruit extracts on cervical cancer (HeLa) and fibroblast (L929) cells. Journal of evidence-based complementary & alternative medicine. 2017;22(4):777-87.

49. Wu S, Tsai J, Chang S, Lin D, Wang S, Huang S, et al. Supercritical carbon dioxide extract exhibits enhanced antioxidant and anti-inflammatory activities of Physalis peruviana. Journal of ethnopharmacology. 2006;108(3):407-13.

50. Gharagozloo M, Jafari S, Esmaeil N, Javid EN, Bagherpour B, Rezaei A. Immunosuppressive effect of silymarin on mitogen‐activated protein kinase signalling pathway: The impact on T cell proliferation and cytokine production. Basic & clinical pharmacology & toxicology. 2013;113(3):209-14.

51. Russo A, Formisano C, Rigano D, Senatore F, Delfine S, Cardile V, et al. Chemical composition and anticancer activity of essential oils of Mediterranean sage (Salvia officinalis L.) grown in different environmental conditions. Food and Chemical Toxicology. 2013;55:42-7.

52. Altindal D, Altindal N. Sage (Salvia officinalis) oils. Essential oils in food preservation, flavor and safety: Elsevier; 2016. p. 715-21.

53. Kamatou GP, Makunga N, Ramogola W, Viljoen AM. South African Salvia species: a review of biological activities and phytochemistry. Journal of ethnopharmacology. 2008;119(3):664-72.

54. Ruckmani A, Meti V, Vijayashree R, Arunkumar R, Konda VR, Prabhu L, et al. Anti-rheumatoid activity of ethanolic extract of Sesamum indicum seed extract in Freund's complete adjuvant induced arthritis in Wistar albino rats. Journal of Traditional and Complementary Medicine. 2018;8(3):377-86.

55. Nahar L. Investigation of the analgesic and antioxidant activity from an ethanol extract of seeds of Sesamum indicum. Pakistan journal of biological sciences: PJBS. 2009;12(7):595-8.

56. Hibasami H, Moteki H, Ishikawa K, Katsuzaki H, Imai K, Yoshioka K, et al. Protodioscin isolated from fenugreek (Trigonella foenumgraecum L.) induces cell death and morphological change indicative of apoptosis in leukemic cell line H-60, but not in gastric cancer cell line KATO III. International Journal of Molecular Medicine. 2003;11(1):23-6.

57. Sindhu G, Shyni G, Pushpan CK, Nambisan B, Helen A. Evaluation of anti-arthritic potential of Trigonella foenum graecum L.(Fenugreek) mucilage against rheumatoid arthritis. Prostaglandins & other lipid mediators. 2018;138:48-53.

58. Pundarikakshudu K, Shah DH, Panchal AH, Bhavsar GC. Anti-inflammatory activity of fenugreek (Trigonella foenum-graecum Linn) seed petroleum ether extract. Indian journal of pharmacology. 2016;48(4):441.

59. Amirghofran Z. Herbal medicines for immunosuppression. Iranian Journal of Allergy, Asthma and Immunology. 2012:111-9.

60. Wang Y, Mei Y, Feng D, Xu L. Triptolide modulates T‐cell inflammatory responses and ameliorates experimental autoimmune encephalomyelitis. Journal of neuroscience research. 2008;86(11):2441-9.

61. Broer J, Behnke B. Immunosuppressant effect of IDS 30, a stinging nettle leaf extract, on myeloid dendritic cells in vitro. The Journal of Rheumatology. 2002;29(4):659-66.

62. Wetherilt H. Evaluation of Urtica species as potential sources of important nutrients. Developments in food science. 29: Elsevier; 1992. p. 15-25.

63. Vetvicka V, Vetvickova J. Immune enhancing effects of WB365, a novel combination of Ashwagandha (Withania somnifera) and Maitake (Grifola frondosa) extracts. North American journal of medical sciences. 2011;3(7):320.

64. Lin B, Zhao Y, Han P, Yue W, Ma X-Q, Rahman K, et al. Anti-arthritic activity of Xanthium strumarium L. extract on complete Freund׳ s adjuvant induced arthritis in rats. Journal of ethnopharmacology. 2014;155(1):248-55.

65. Chopra R, Nayar S, Chopra I. Glossary of Indian Medicinal Plants Council of Scientific and Industrial Research. New Delhi. 1956;89.

66. Srivastava K, Mustafa T. Ginger (Zingiber officinale) and rheumatic disorders. Medical hypotheses. 1989;29(1):25-8.
