# Supplementary material for: Discovery of potential natural dihydroorotate dehydrogenase inhibitors and their synergism with brequinar via integrated molecular docking, dynamic simulations and in vitro approach
Source: Sci Rep. 2022 Nov 9;12:19037. doi: 10.1038/s41598-022-23006-1 (PMC9646789; doi:10.1038/s41598-022-23006-1)
Supplement: Supplementary file 4 — Supplementary Information 4. [file 41598_2022_23006_MOESM4_ESM.pptx]

## Slide 1
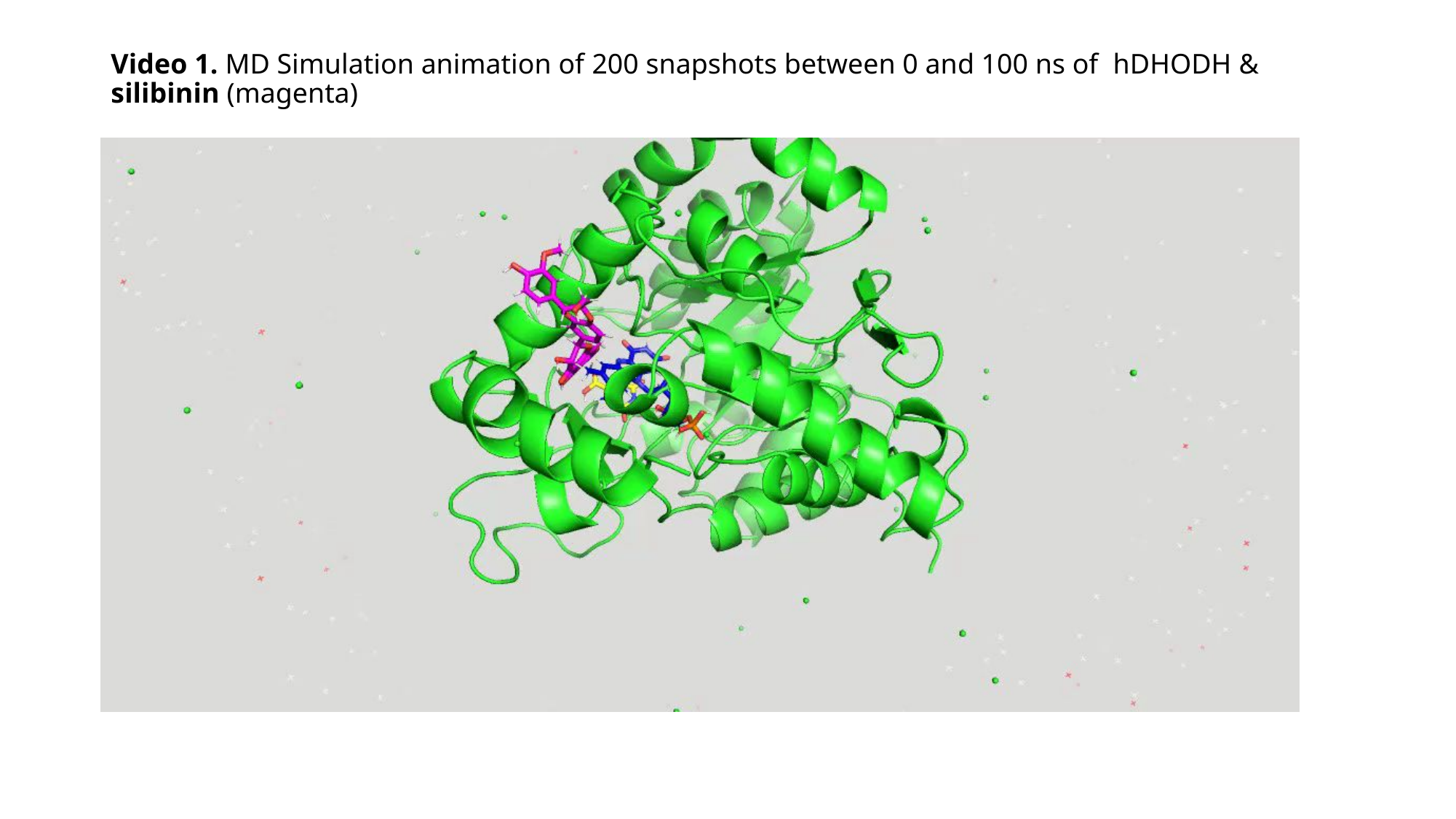

# Video 1. MD Simulation animation of 200 snapshots between 0 and 100 ns of hDHODH & silibinin (magenta)

## Slide 2
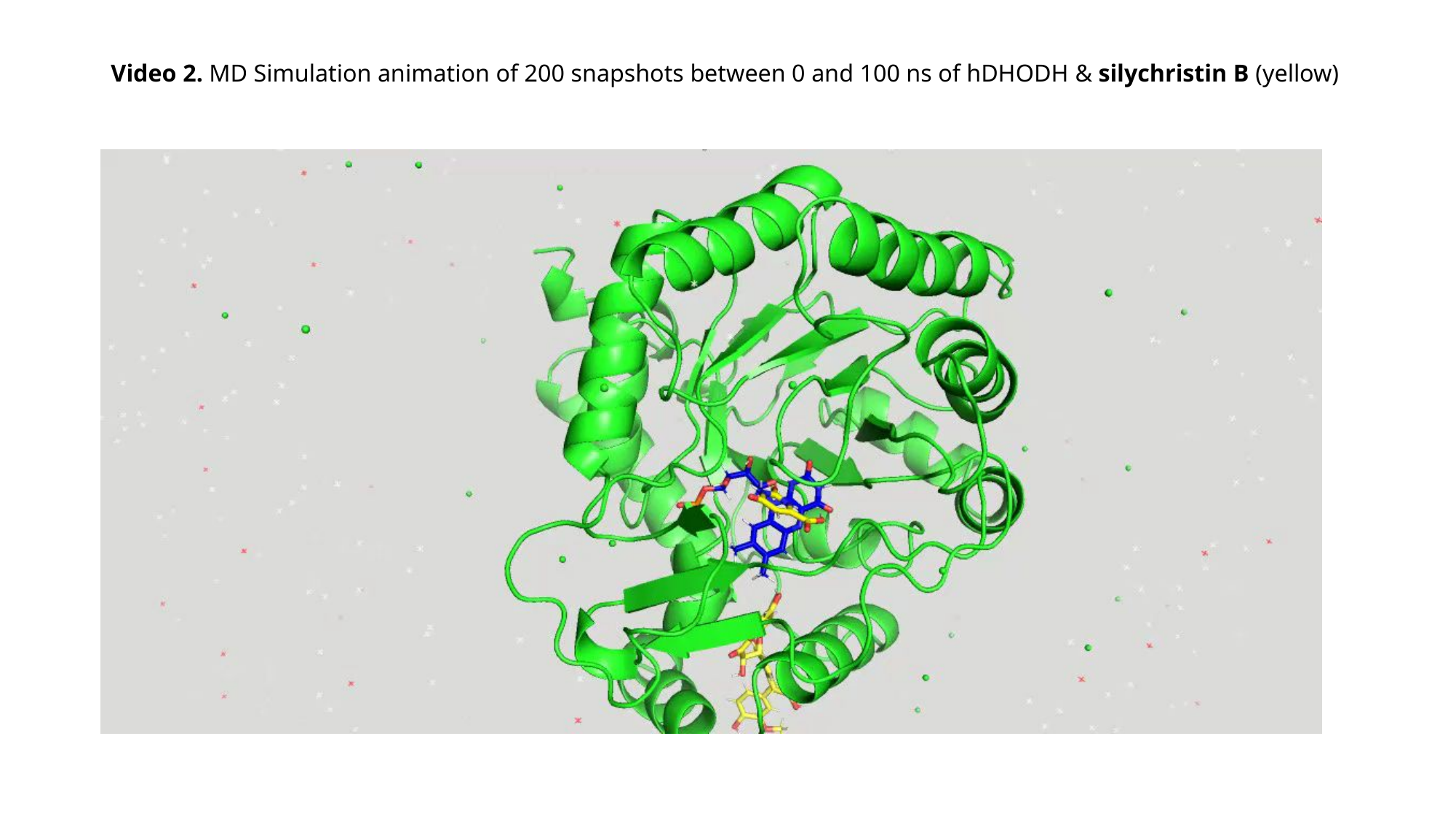

# Video 2. MD Simulation animation of 200 snapshots between 0 and 100 ns of hDHODH & silychristin B (yellow)

## Slide 3
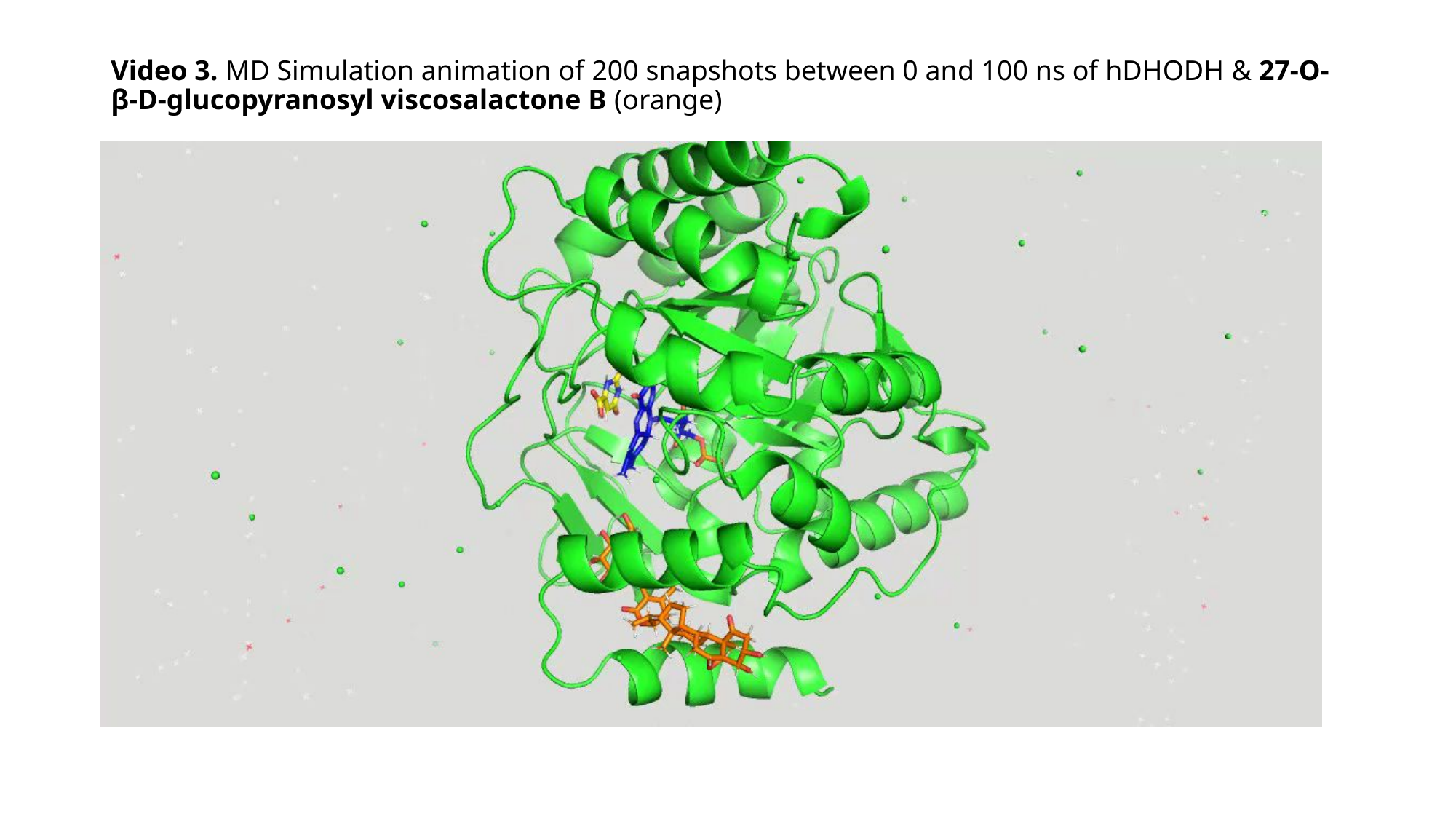

# Video 3. MD Simulation animation of 200 snapshots between 0 and 100 ns of hDHODH & 27-O-β-D-glucopyranosyl viscosalactone B (orange)

## Slide 4
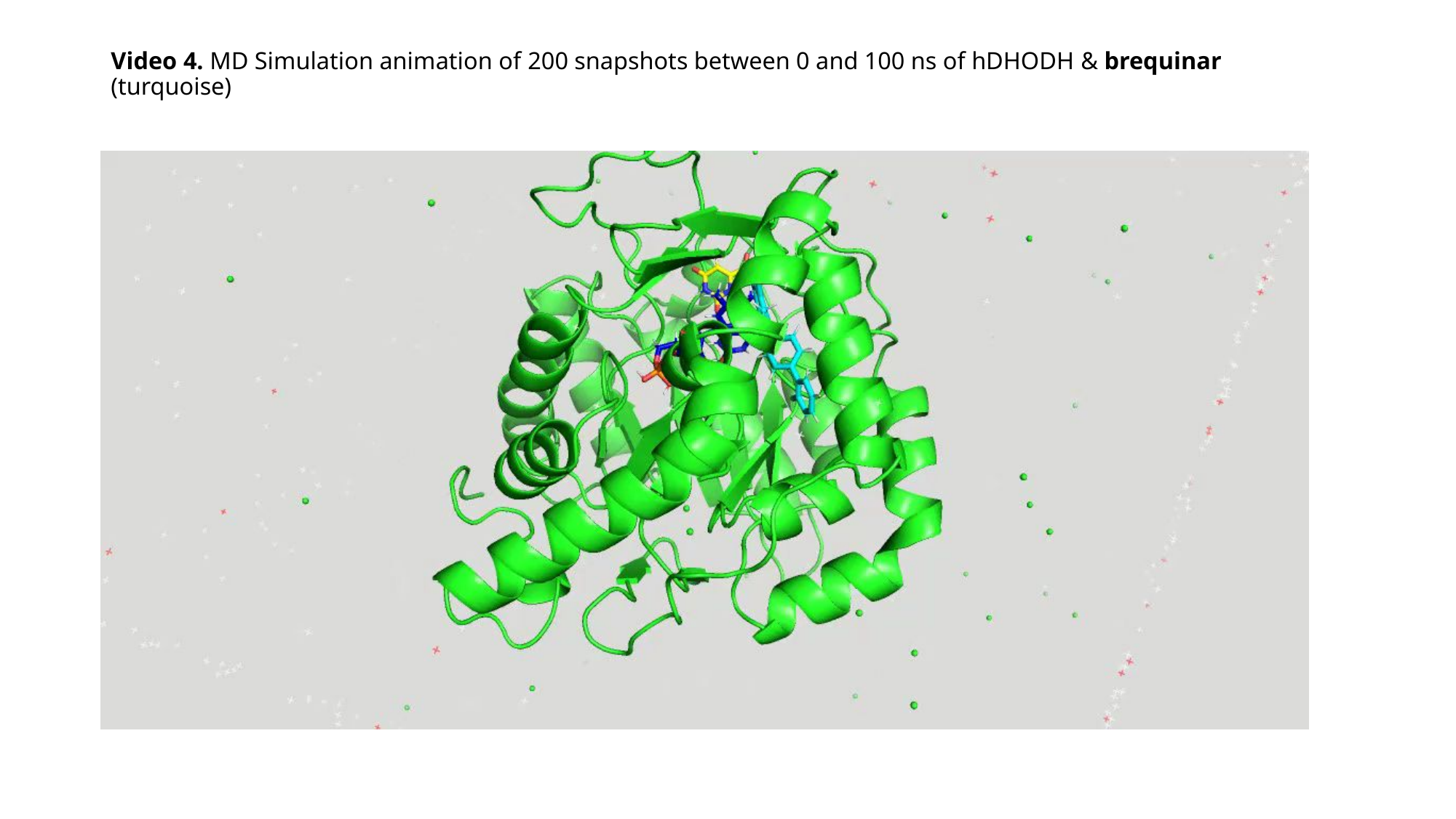

# Video 4. MD Simulation animation of 200 snapshots between 0 and 100 ns of hDHODH & brequinar (turquoise)
